# Protein nanovaccine confers robust immunity against *Toxoplasma*

**DOI:** 10.1038/s41541-017-0024-6

**Published:** 2017-09-05

**Authors:** Kamal El Bissati, Ying Zhou, Sara Maria Paulillo, Senthil Kumar Raman, Christopher P. Karch, Craig W. Roberts, David E. Lanar, Steve Reed, Chris Fox, Darrick Carter, Jeff Alexander, Alessandro Sette, John Sidney, Hernan Lorenzi, Ian J. Begeman, Peter Burkhard, Rima McLeod

**Affiliations:** 1 Departments of OVS, The University of Chicago, 5841S Maryland Ave, Chicago, IL 60637 USA; 2 Alpha-O Peptides AG, Lörracherstrasse 50, 4125 Riehen, Switzerland; 3 Institute of Materials Science and Department of Molecular and Cell Biology, University of Connecticut, 97 North Eagleville Road, Storrs, CT 06269 USA; 4 Strathclyde Institute of Pharmacy and Biomedical Sciences, University of Strathclyde, Glasgow, G4 0RE UK; 5 Walter Reed Army Institute of Research, 503 Robert Grant Ave, Silver Spring, MD 20910 USA; 6 Infectious Diseases Research Institute, 1616 Eastlake Ave E #400, Seattle, WA 98102 USA; 7 PaxVax, 3985-A Sorrento Valley Blvd, San Diego, CA 92121 USA; 8 La Jolla Institute of Allergy and Immunology, 9420 Athena Cir, La Jolla, CA 92037 USA; 9 J. Craig Venter Institute, 9714 Medical Center Drive, Rockville, MD 20850 USA; 10 Pediatrics (Infectious Diseases), The University of Chicago, 5841S Maryland Ave, Chicago, IL 60637 USA

## Abstract

We designed and produced a self-assembling protein nanoparticle. This self-assembling protein nanoparticle contains five CD8^+^ HLA-A03-11 supertypes-restricted epitopes from antigens expressed during *Toxoplasma gondii*’s lifecycle, the universal CD4^+^ T cell epitope PADRE, and flagellin as a scaffold and TLR5 agonist. These CD8^+^ T cell epitopes were separated by N/KAAA spacers and optimized for proteasomal cleavage. Self-assembling protein nanoparticle adjuvanted with TLR4 ligand-emulsion GLA-SE were evaluated for their efficacy in inducing IFN-γ responses and protection of HLA-A*1101 transgenic mice against *T. gondii*. Immunization, using self-assembling protein nanoparticle-GLA-SE, activated CD8^+^ T cells to produce IFN-γ. Self-assembling protein nanoparticle-GLA-SE also protected HLA-A*1101 transgenic mice against subsequent challenge with Type II parasites. Hence, combining CD8^+^ T cell-eliciting peptides and PADRE into a multi-epitope protein that forms a nanoparticle, administered with GLA-SE, leads to efficient presentation by major histocompatibility complex Class I and II molecules. Furthermore, these results suggest that activation of TLR4 and TLR5 could be useful for development of vaccines that elicit T cells to prevent toxoplasmosis in humans.

## Toxoplasmosis: a pioneering vaccine for the most common parasitic infection

*Toxoplasma gondii* is the most common parasitic infection worldwide. It causes brain and eye damage as well as death. The organism is present in the brain of approximately two billion people worldwide. Although active infection can be treated, there is no cure. A vaccine is greatly needed. Herein, we demonstrate that we can produce a self-assembling protein nanoparticle (SAPN) with flagellin as a TLR5 ligand immune stimulating signal, and as a scaffold for CD8+ and CD4+ T cell-eliciting epitopes. We find this immunogen confers robust protection in our human HLA class I transgenic mouse model. Our SAPN contains peptides that elicit production of IFN-γ by CD8+ T lymphocytes from transgenic mice, as well as by CD4+ T cells. The SAPNs are further adjuvanted with a TLR4 ligand. Our adjuvanted, immunosense SAPN that combines stimuli for innate and adaptive immunity, presents an advancement in vaccine development that has considerable promise for protection against infections.

## Introduction

*Toxoplasma gondii* infects all mammals. It can cause severe brain and eye damage in the fetus, in newborn infants, and in immune-compromised individuals.^1^ Although anti-parasitic medicines such as sulfadiazine and pyrimethamine are available, some patients experience side effects including toxicity and hypersensitivity. Latent, encysted parasites are not eliminated by these treatments.^2^ Therefore, development of a potent, safe, effective vaccine is greatly needed.

One approach for toxoplasmosis vaccine development is an epitope-based vaccine designed to enhance host immunity. Protection is achieved through stimulation of CD4^+^ helper T lymphocytes and CD8^+^ IFN-γ producing T lymphocyte responses. These CD8^+^ T cells recognize octamer/nonamer peptides presented on HLA supermotif molecules on infected cells. Previously, our laboratory (RM, KE) identified epitopes eliciting CD8^+^ T cells derived from proteins expressed during different phases of the *Toxoplasma* life cycle. HLA-A02, A03-11 and B07 human, supermotif, major histocompatibility complex (MHC) molecules are present in ~90% of humans,^3–6^ and therefore are capable of presenting these epitopes. As the discovery of such protective peptide epitopes accumulates, mechanisms are needed to effectively present these epitopes to the immune system of the host.

We have pioneered a platform known as Self-Assembling Protein Nanoparticles (SAPNs).^7–12^ SAPNs induce a strong immune response due to the repetitive display of antigens.^7, 10, 12^ They promote immune responses by CD4^+^ as well as CD8^+^ T cells by incorporating the T cell epitopes into the core architecture of the nanoparticle.^8, 9, 11^ They trigger a strong innate immune response by activating the TLR5 pathway through the adjuvant flagellin.^13^ Because of their size and shape they have the potential to reach follicular dendritic cells that are critical for antigen presentation and processing.^14^ Although macrophages play a role in immunity, interactions between SAPN and macrophages were not studied. SAPNs induce immune response that are orders of magnitudes stronger than Keyhole limpet hemocyanin, which is a standard vaccine carrier. We previously designed SAPN-based vaccine candidates for various infectious diseases including malaria,^10, 11, 14, 15^ HIV,^16^ SARS,^17^ and influenza.^18^

**Fig. 1:**
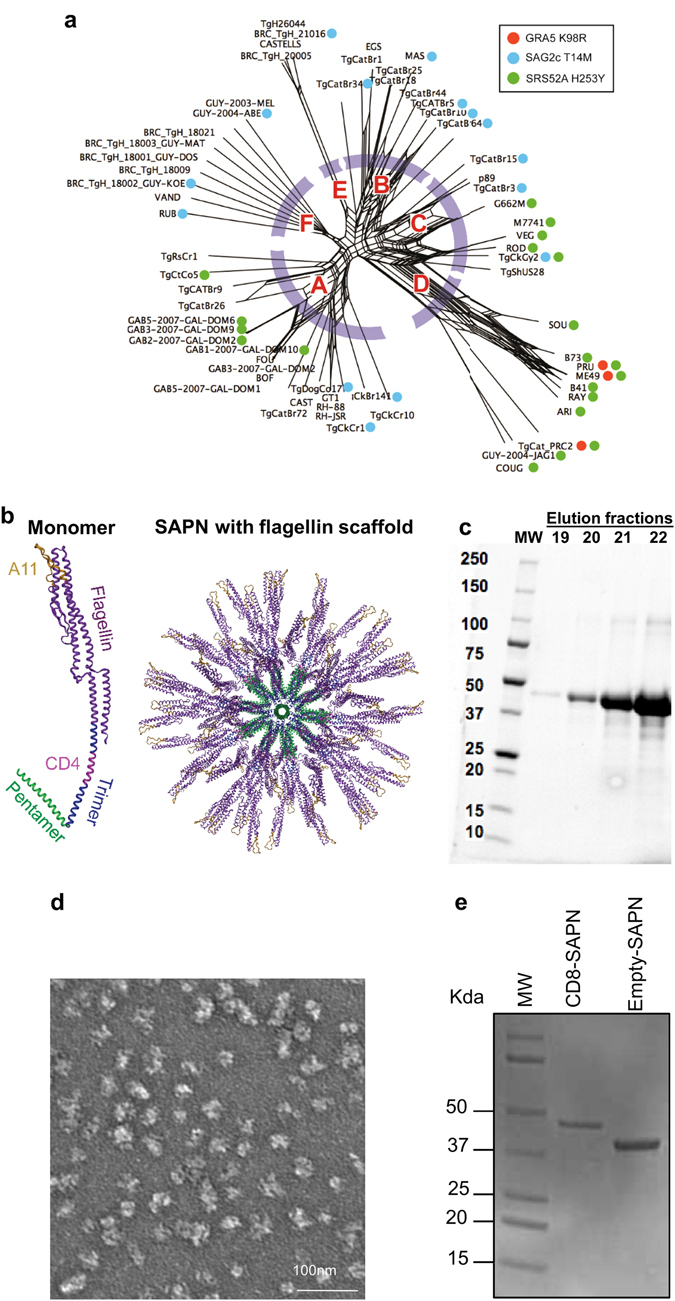
Assembly of *CD8-SAPNs*. **a** Phylogenetic tree showing 62 genetic isolates of *Toxoplasma* analyzed herein. These are in the multi-sequence alignments of proteins, and peptides derived from them, utilized to create our artificial immunogenic (“smart”) protein. **b** Flagellin is used as a scaffold into which epitopes are intercalated from *Toxoplasma*. Earlier logic for inclusion of flagellin as adjuvant and scaffold came from work with malaria (http://www.internationalinnovation.com/build/wp-content/uploads/2016/05/David_Lanar_Intl_Innovation_Infectious_Diseases_Research_Media_LR.pdf),^19^ as well as with influenza.^24^ Computer model of the prototype. The core particle composed of the pentameric and trimeric coiled coils is shown in green and blue, respectively. The following are attached to the trimeric coiled coil: the TRL5 agonist flagellin (D0 and D1 domains) (purple) with the A11 epitopes (yellow) and a CD4 epitope string (magenta). **c** SDS-PAGE of the purified protein. Lanes are as follows: Lane 1: MW (molecular weight markers). Lane 2–5: Elution fractions were from 19 to 22. Samples derive from the same experiment and the gels/blots were processed in parallel. **d** Transmission electron microscopy of the nanoparticle preparation. **e** SDS-PAGE 4–20% of the purified protein. Lane 1: MW (molecular weight markers). Lane 2: *CD8-SAPN*. Lane 3: *Empty-SAPN*. Samples derive from the same experiment and the gels/blots were processed in parallel

Earlier findings, and recent parallel work with a recombinant polypeptide, SAPNs, and GLA-SE (Fig. 1 and unpublished data [DL]) provide the foundation for our present studies. These earlier findings provide a basis for use of immunosense selected peptides from different genetic isolates of *T. gondii* (Fig. 1a), a flagellin scaffold,^7, 8, 13, 19^ and adjuvanting with GLA-SE.^20–23^ Earlier studies from the Walter Reed Army Institute of Research with malaria based SAPNs demonstrated that flagellin molecules improved immunogenicity (DL, PB, unpublished work). Initially, this was the basis for using flagellin as a SAPN scaffold in our *T. gondii* studies (Fig. 1b). This approach was also used in our work with influenza.^24^ This work suggested that flagellin would be helpful as a scaffold and immunogen in our newest *T. gondii* work.

**Table 1:** Rationale for construction of immunogenic preparation: Summary of published preclinical in vivo comparisons of GLA-SE, SE, and GLA-AF

Formulations tested	Vaccine antigen	Animal model	Immunization route	GLA dose (μg)	Summary of comparative findings	Reference
SE, GLA-AF, GLA-SE	LmSTI1 (Leishmaniasis)	Mouse (BALB/c)	Subcutaneous	20	GLA-SE elicited higher IgG2a/IgG1 antibody ratio compared to GLA-AF or SE	19
SE, GLA-AF, GLA-SE	PfCelTOS (Malaria)	Mouse (BALB/c)	Subcutaneous	5	GLA-SE and GLA-AF elicited similar IgG1 and IgG2a antibody titers but higher IgG2a titers compared to SE; only GLA-SE enhanced IFN-γ and IL-17 levels compared to antigen alone whereas SE elicited IL-5	20
SE, GLA-AF, GLA-SE	Fluzone® (Influenza)	Mouse (BALB/c)	Intramuscular	20	GLA-SE induced higher IgG2a antibody titers compared to GLA-AF, and both GLA-AF and GLA-SE induced higher IgG2a than SE; SE and especially GLA-SE elicited enhanced HI titers compared to GLA-AF, and GLA-SE induced higher IFN-γ and IL-2 production compared to GLA-AF or SE	21
GLA-AF, GLA-SE	ID93 (Tuberculosis)	Mouse (C57BL/6)	Intramuscular	5	GLA-SE and GLA-AF enhanced IgG2a antibody titers to similar levels; GLA-SE, but not GLA-AF, induced significant levels of Th1-type cytokines (IFN-γ, TNF-α, IL-2); only GLA-SE provided protection from TB challenge	22

**Table 2:** Rationale for construction of immunogenic preparation: Multisequence alignment of octamer/nonamer epitopes demonstrates conservation and variability^a^

^a^ Current haplogroups are shown in column 2. Gray and absence of shading shows demarcation between the haplogroups. The bolded values show the octamer/nonamer epitopes. The red letters show variability in those octamer/nonamer epitopes

**Table 3:** Rationale for construction of immunogenic preparation: Number of isoforms identified per protein or oligopeptide for GRA5, GRA6, SAG1, SAG2C, and SRS52A encoding genes

	GRA5	GRA6	SAG1	SAG2C	SRS52A
Protein	10	14	8	13	9
Oligopeptide	2	0	0	2	2

**Table 4:** Rationale for construction of immunogenic preparation: Predicted binding affinity of worldwide octamer/nonamers

				Predicted IC50 nM				Stability prediction		
Pair	Origin	Peptide	Length	Median	ANN	SMM	NetMHCpan	Pred	Thalf(h)	%Rank_Stab
1	SAG2C (13–21)	STFWPCLLR	9	13	13	19	10	0.846	4.15	0.50
1		SMFWPCLLR	9	18	17	40	18	0.309	0.59	4.00
2	SRS52A (250–258)	SSAHVFSVK	9	14	14	18	7	0.806	3.22	0.70
2		SSAYVFSVK	9	13	13	19	8	0.739	2.30	0.90
3	GRA5 (89–98)	AVVSLLRLLK	10	17	18	15	17	0.948	12.86	0.12
3		AVVSLLRLLR	10	128	81	128	132	0.730	2.20	1.00

In experiments that provided a significant part of the foundation for our approach with SAPN to protect against toxoplasmosis, we (DL, PB, unpublished work) found the following: 1) GLA-SE or GLA-SE-like adjuvant was needed to produce significant titers of anti-nanoparticle antibody; 2) Purified IgG from immunized monkeys completely protected naïve mice (100%), when they were challenged with a lethal dose of 5000 sporozoites that express full-length *Plasmodium. falciparum* Circumsporozoite protein. Purified IgG from a control monkey did not protect any mice; 3) Purified IgG from immunized monkeys, mixed with *P. falciparum* sporozoites, prevented the sporozoite from infecting primary hepatocytes from human liver in tissue culture. IgG from control monkeys did not. Thus, we used this preliminary, foundational data when we chose GLA-SE as the adjuvant for our studies herein. GLA-SE has two components, GLA and SE. GLA is too hydrophobic to be used alone and any formulation of GLA would have other excipients making the formulation nonequivalent to GLA. Earlier studies demonstrated that the emulsion, called “SE”, did not adjuvant most proteins when administered alone. At present, GLA-SE is in pre-clinical studies or clinical trials as an adjuvant to prevent cancer, herpes, *Leishmania*, and *Mycobacterium tuberculosis* infections. Our earlier studies also demonstrated that GLA-SE was superior to ALUM as an adjuvant for our polypeptide.^25^ GLA-SE was also superior to ALUM in primates immunized with SAPN. In fact, ALUM diminished the response to GLA-SE plus SAPN (DL, PB, unpublished work).

In our previous studies with *T. gondii*, we constructed SAPNs displaying the dense granule epitope (GRA7_20–28_) and pan-DR binding epitope PADRE.^26^ We evaluated these vaccine components in HLA-B*0702 transgenic mice.^9^ Immunization of these mice activated GRA7-specific CD8^+^ T cells that produced IFN-γ. Thereby, these mice were protected against subsequent challenge with high inocula of Type I and Type II parasites. These initial results highlighted the potential to protect against toxoplasmosis with a SAPNs vaccine approach.

In the present study, five epitopes which bind to HLA-A11-01 were evaluated for their efficacy in a SAPN-vaccine in HLA-A11-01 transgenic mice.^5^ These included epitopes from the surface antigen (SAG1), the dense granule proteins (GRA5 and GRA6), and the surface antigen-1-related sequences (SRS52A).^5^ In these constructs, the CD8^+^ HLA-A03-11 supertypes-restricted epitopes were linked by N/KAAA spacers. They were conjugated with PADRE, a universal CD4^+^ helper T lymphocyte epitope.^26^ This synthetic polypeptide is effective in mice and more effective than the pooled peptides separately.^5, 23^ PADRE binds promiscuously to MHC class II variants, and augments effector functions of CD8 + T cells through stimulation of IL2 production by CD4^+^ T helper cells.^27, 28^ Epitopes eliciting both CD4^+^ and CD8^+^ T cells are important components in the formulation of successful vaccines that drive protective responses.^29^ Our data show that incorporating PADRE into the SAPN constructs and delivering it in TLR4 ligand emulsion adjuvant (GLA-SE), resulted in activation of CD8^+^ T cells. This vaccine formulation led these cells to produce IFN-γ. They protected against subsequent challenge with Type II parasites given as a high inoculum. Thus, our work highlights the potential for the use of SAPN as a platform for the delivery of CD8^+^ and CD4^+^-restricted epitopes, formulated with the GLA-SE adjuvant, to protect against toxoplasmosis.

## Results

### Preparation and characterization of CD8^+^-SAPN and empty-SAPN

The SAPN constructs were expressed, purified and folded to form nanoparticles (Fig. 1c–e). The protein has a relative molecular weight of about 48 kDa on a Sodium dodecyl sulfate polyacrylamide gel electrophoresis (SDS-PAGE) (Fig. 1c, e). Transmission electron microscopy (Fig. 1d) showed a relatively uniform distribution of non-aggregated nanoparticles of about 30 nm in diameter.

### In vivo immunogenicity of CD8^+^ T cell-eliciting SAPNs

**Fig. 2:**
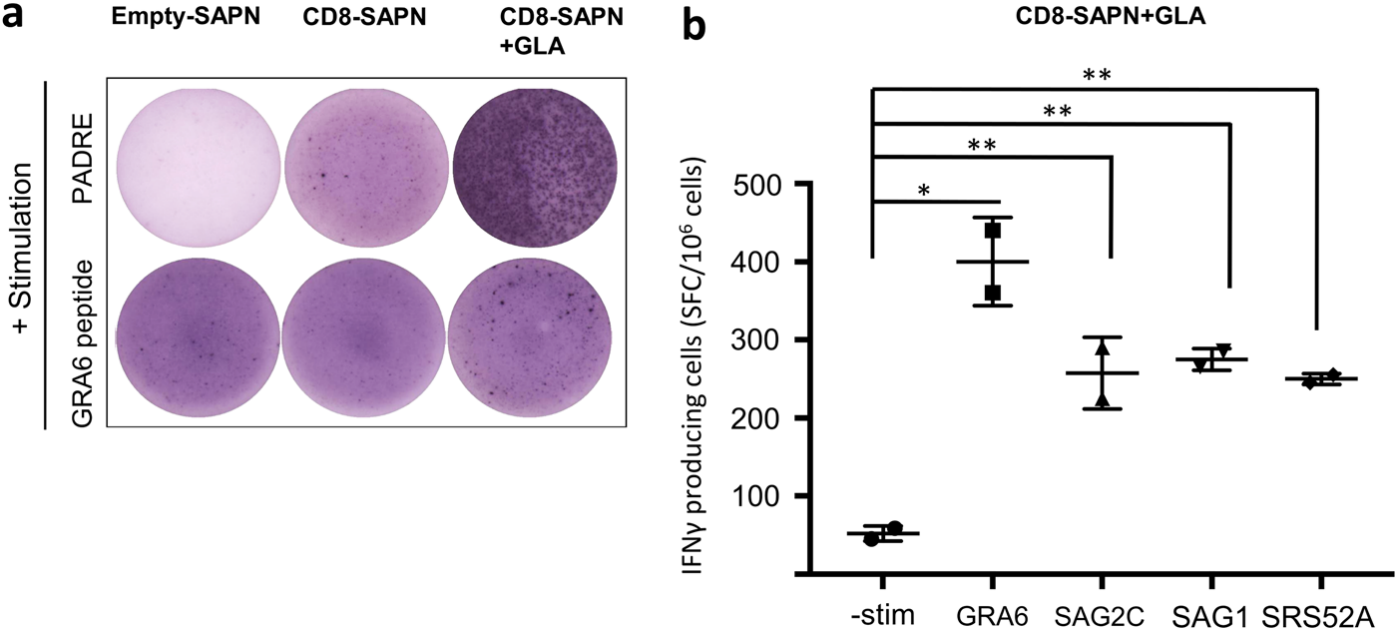
*CD8-SAPNs* elicit restricted CD8^+^ T and CD4^+^ T cell peptide-specific immune response. ELISpot showing IFN-γ spot formation. Mouse splenocytes from *Empty-SAPN*, *CD8-SAPN*, and *CD8-SAPN + GLA-SE* were tested using GRA6 peptide (GRA6_164–172_) or PADRE. GLA designates GLA-SE in this figure. All peptides elicited IFN-γ (*p* < 0.05) compared to unstimulated cultures. Pooled peptides^25^ appeared additive. The greatest effect occurred with the polypeptide as also occurred in earlier studies.^25^ *p < 0.05

**Fig. 3:**
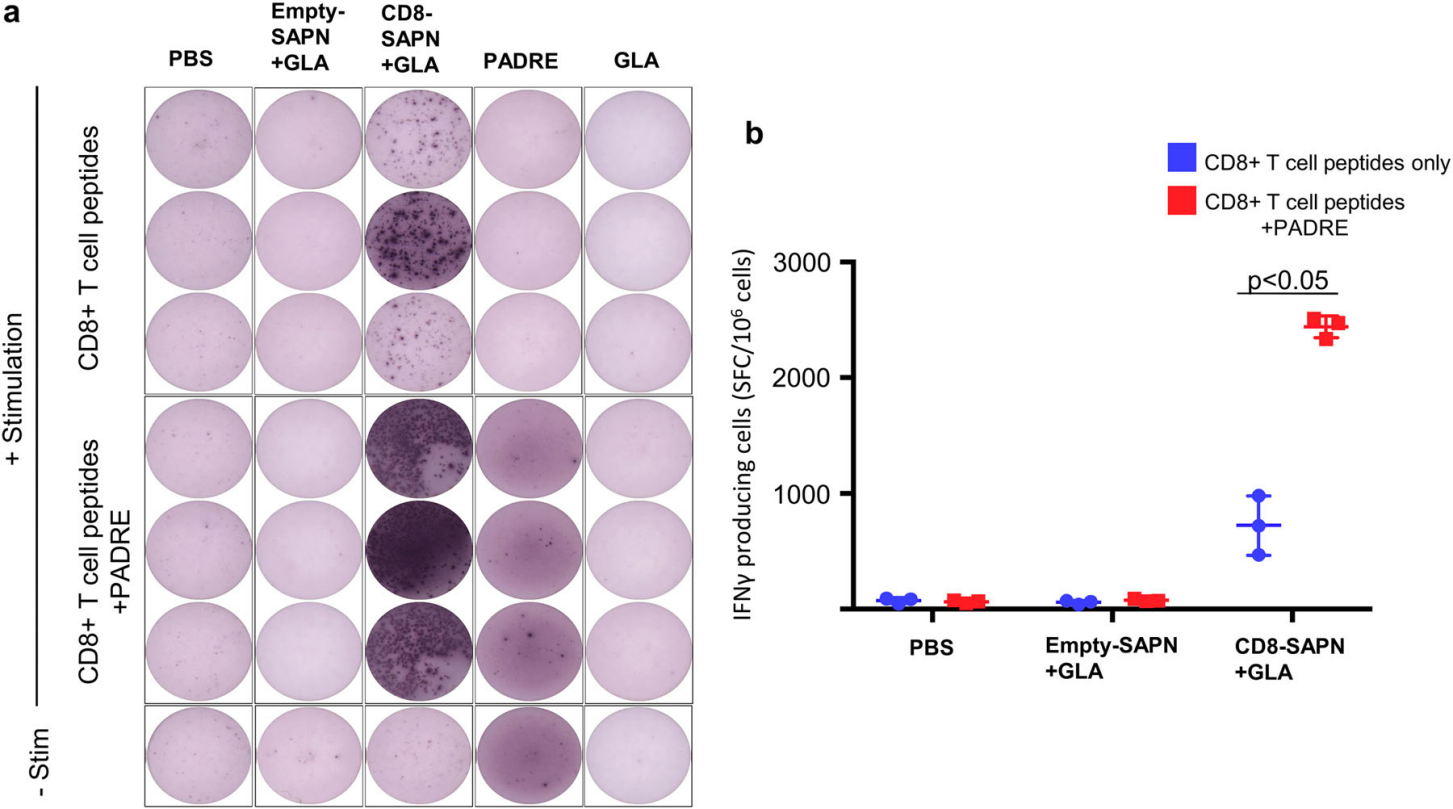
*CD8-SAPNs* are potent inducers of cell-mediated immunity. **a** IFN-γ ELISpot assay stimulated with a group of 5 peptides HLA-A*1101. **b** Graph shows the count of spots for splenocytes of untreated, *Empty-SAPN* + GLA1 *CD8-SAPN + GLA-SE* group of mice. GLA designates GLA-SE in this figure. *p < 0.05 for all IFN-γ ELISpots compared to controls

HLA-A*1101-transgenic mice were immunized intramuscularly with CD8^+^ T cell-eliciting SAPNs combined with GLA-SE. Mice were immunized three times intramuscularly at 2 week intervals. Empty-SAPNs plus GLA-SE or PBS were used in sham immunizations of control mice. CD8^+^-T cell-eliciting SAPN-GLA-SE vs. Empty-SAPN-GLA-SE were compared in HLA-A*1101 transgenic mice as described. Spleen cells were obtained from immunized HLA-A*1101 transgenic mice 2 weeks after final immunization. IFN-γ produced by splenocytes cultured with the pool of peptides was measured. Figure 2 shows IFN-γ secretion is high in mice immunized with CD8^+^-T cell-eliciting SAPN plus GLA-SE when stimulated with PADRE or the GRA6 peptide. The other peptides also elicited IFN-γ production. In our earlier work,^25^ and herein, effects of the separate peptides were additive (Figs. 2b and 3). The polyepitopes elicited the best response earlier^25^ and herein (Fig. 3). Figure 3a and b indicate that IFN-γ secretion in cultures with the SAG1, GRA6, GRA3, and SRS52A peptides was significantly enhanced by immunization with these peptides but not Empty-SAPN or PBS. Significantly more IFN-γ secretion was observed when cells were stimulated with these pooled peptides plus PADRE. Thus, the association of CD8^+^ T cell- and CD4^+^ T cell-restricted peptides contributes to IFN-γ production in HLA-A*1101 transgenic mice.

### In vitro TLR5 stimulation

**Fig. 4:**
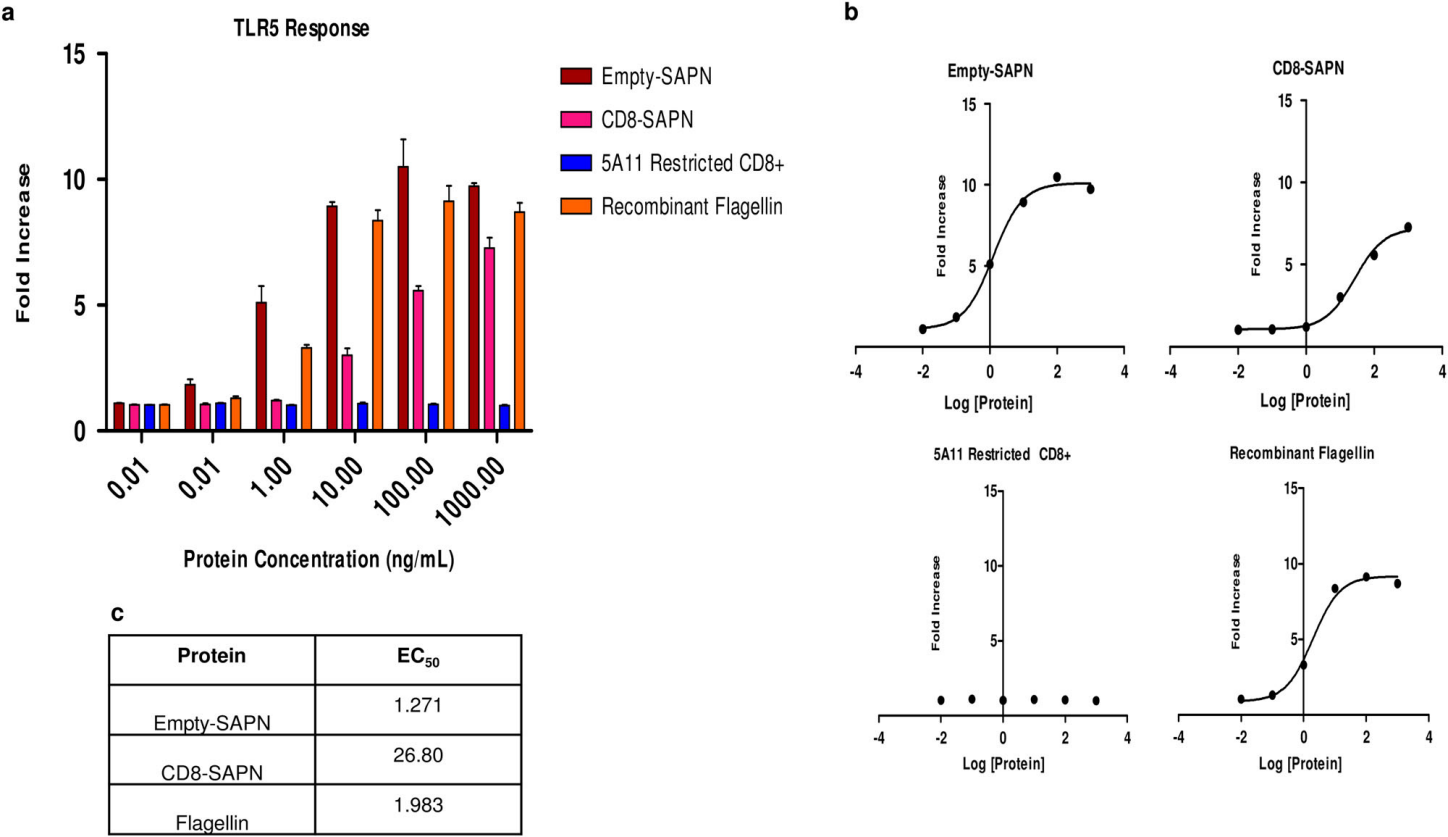
Seaporter TLR5 cell line responses to flagellin and SAPN. SeaPorter TLR5 cell-line was exposed to varying concentrations of each indicated protein, and the level of TLR5 stimulation was determined by the level of SEAP expression. Fold increase in SEAP expression for each protein sample over non-treated controls, error bars are standard error of the means. A two-way ANOVA model was fit with protein concentration and type as factors. There was a significant protein concentration by type interaction (*p* < 0.001); this indicated that the differences across types depended on the concentration and that the differences across concentrations varied by type. Specifically, there weren’t statistically significant differences across types at the two lowest concentrations (0.01 and 0.1), but there were significant differences between types at the 1, 10, 100, and 1000 ng/mL concentrations (*p* < 0.001 for all). Subsequent pairwise contrasts at these 4 concentrations found that the 5A11 Restricted CD8 + group (the recombinant protein without flagellin) was significantly different from the other 3 groups in all cases except for the 5A11 Restricted CD8 + vs. CD8-SAPN comparison at the 1 ng/mL concentration. In addition, at each of these 4 concentrations, the Empty-SAPN was significantly different (greater than) from the CD8-SAPN. There was a significant concentration effect for all protein types (*p* < 0.001) except 5A11 Restricted CD8 + (*p* > 0.9)

The SeaPorter TLR5 cell-line was exposed to varying concentrations of the SAPNs. The SAPN included: Empty-SAPN that do not contain the CD8^+^ epitopes but still have flagellin; CD8^+^-SAPN containing the polypeptide with the five restricted CD8^+^ epitopes; recombinant polypeptide; and recombinant flagellin (as control). The concentrations of SAPNs used were 0.01, 0.1, 1, 10, 100, and 1000 ng/ml. Fold increase in SEAP expression for each protein sample over non-treated controls reflected level of TLR5 stimulation. As shown in Fig. 4a–c, TLR5 activity was significantly enhanced by the Empty-SAPNs and the CD8^+^-T cell-eliciting SAPNs, but not the control polypeptide. Surprisingly, flagellin in Empty-SAPN particles have higher TLR5 activity than recombinant flagellin alone.

### SAPNs with GLA-SE adjuvant confer robust protection against *T. gondii* in HLA-A*1101 transgenic mice

**Fig. 5:**
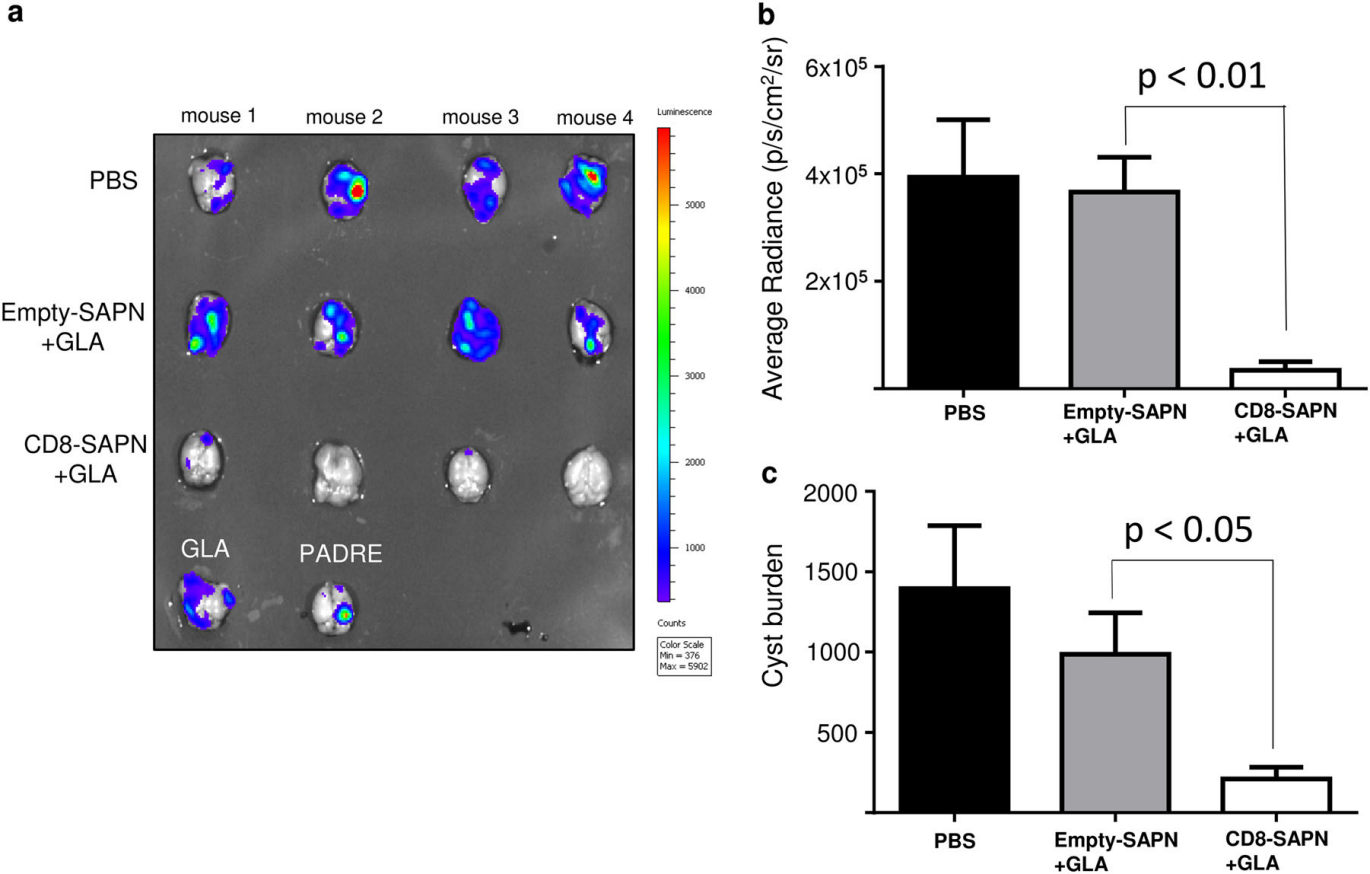
*T. gondii* brain cysts luciferase expression was significantly reduced in immunized HLA-A*1101 mice. HLA-A*1101 transgenic mice were immunized with GLA-SE adjuvanted *Empty-SAPN* or *CD8-SAPN* three times at intervals of 2 weeks. PBS was used as control. **a** *T. gondii* brain cysts luciferase expression was significantly reduced in HLA-A*1101 mice immunized with *CD8-SAPN* plus GLA boost at 21 days after challenge with 2000 Me49 (Fluc) *T. gondii* expressing luciferase. **b** Xenogen imaging of brain *ex vivo* following the injection of luciferin into the retro-orbital plexus and then exposure of the brain to luciferin solution. This figure shows data from mice in one of the replicate experiments (*n* = 4 control and 4 immunized mice). **c** Enumeration of cyst was performed with brains of mice challenged 21 days after final immunization. SAPN reduced cyst numbers and luminescence (*p* < 0.05)

In the results shown in Fig. 5, we had immunized mice with either CD8^+^ T cell-eliciting SAPN with GLA-SE adjuvant, or Empty-SAPN with GLA-SE adjuvant, or adjuvant alone, or PADRE alone, or PBS. We then challenged 2 weeks after the last immunization with Type II strains of *T. gondii* expressing luciferase. Brains from these mice were imaged with a Xenogen camera 21 days after challenge with 2000 Me49-Fluc tachyzoites. Figure 5a and b show that luminescence from *T. gondii* in mice immunized with CD8^+^ T cell-eliciting SAPN plus GLA-SE was significantly lower than in mice immunized with control Empty-SAPN plus GLA-SE, GLA-SE alone, PADRE alone, or PBS. This finding correlates with a reduction of the number of cysts per brain in mice that received CD8^+^-T cell-eliciting SAPN plus GLA-SE adjuvant (Fig. 5c).

## Discussion

Improved vaccination and delivery approaches to elicit cellular immune responses against *T. gondii* are needed. In our previous studies we defined a panel of octamer/nonamer peptides restricted by MHC class I molecules. These peptide epitopes bind to and elicit IFN-γ responses from CD8^+^ T cells isolated from HLA A02, A03, and B07 individuals. These class I supermotifs are present in essentially all the human population worldwide, but with different frequency in different regions. When given with the GLA-SE adjuvant, these pooled peptides were able to protect haplotype specific HLA supermotif transgenic mice. This protection was measured as survival and reduced parasite burden.

Our capability to control the ability of peptides and proteins to self-assemble into particles which have a well-defined size and shape allows us to design mechanically and chemically stable particles. These SAPNs combine strong immunogenic effects of live attenuated vaccines with high specificity in eliciting immune responses of protein-based vaccines because they resemble virus capsids. It is apparent that the SAPNs have a great potential to serve as a platform for vaccines beyond their ability to present antigens in a repetitive manner. In contrast to live attenuated vaccines, SAPN-derived vaccines pose no significant risk of infection. They are very versatile and flexible in their design leading to better biophysical and immunologic properties. Furthermore, bacterial protein expression, purification, and self-assembly into nanoparticles reduces the time needed for large-scale vaccine production.

Herein, we used the SAPNs to present immunogenic peptide epitopes to a host’s immune system based on the assembly of five protective CD8^+^ CTL HLA-A03-11 restricted supertypes in addition to the universal helper epitope, PADRE. All epitopes were flanked at the C-terminus by N/KAAA spacers, which promote optimal immunogenic processing. Our data showed potent immunogenicity (high IFN-γ secretion) when splenocytes were stimulated by these peptides through immunization in vivo, and then exposure in vitro. In separate studies,^24^ we found that SAPN, which contains flagellin, protected better against influenza than SAPN without flagellin. This flagellin scaffold then became our SAPN platform going forward. In our TLR5 activity assay, the SAPN with the flagellin scaffold shows good stimulation of TLR5. However, the activity is reduced compared to the Empty-SAPN. This could be due to some interference with TLR5-binding and the presentation of the CD8^+^ T cell-restricted epitopes because the CD8^+^ epitopes string was engineered into the flagellin molecule to replace the D2 and D3 flagellin domains.

Thus, our future work will utilize this approach to engineer different SAPN constructs with optimized processing and immunogenicity for all our vaccine constituents. The proposed mechanisms for inducing innate immunity by our SAPN is the ligation of TLR4 by GLA in an emulsion^30^ and TLR5 by flagellin on the surface of the SAPN.^13^ McCoy et al.’s data suggested cross presentation^14^ of CD8^+^ stimulating epitopes in SAPNs (Fig. 1a). GLA-SE has been used with SAPN to successfully immunize against *P. falciparum* by eliciting antibody and T cells, whereas SAPN without GLA-SE was not effective (DL, PB, unpublished results). The adjuvant was safe in primates and now is entering clinical trials in humans. Despite remarkable protection provided by our SAPN vaccination in this study, some brain cysts were still detected. Thus, potential improvements in induction of protective immune responses could be made with the addition of separate nanoparticles with other CD4^+^ and CD8^+^ T cell-eliciting epitopes of various *T. gondii* proteins from several parasite life stages and potentially B-cell epitopes to stimulate a potent antibody response. Cell-mediated immunity, with cytolytic T cells and IFN-γ production, is considered to be the desired primary, protective, immune response.^28, 31^ Nonetheless, antibodies may contribute to protection. Addition of the micronemal proteins (MICs) or other proteins that induce antibodies that are neutralizing, adhesion or invasion blocking, or complement fixing, could further improve protection, if they play a significant part in attachment to or penetration of the host cell by the parasite. MICs have recently been used as recombinant vaccines and showed promising protection levels.^32^ MIC1 also stimulates IL 12 production in mice. Possibly, these proteins could also be engineered in separate SAPNs to yield a multi-SAPN vaccine to protect against toxoplasmosis.

Earlier studies provide support for using GLA-SE as an adjuvant for a wide variety of protein vaccines, including our own. We evaluated a *P. falciparum* SAPN vaccine and demonstrated GLA-SE was essential, or improved immunogenicity, vs. a related SAPN (DL, PB, unpublished). This study involved presenting antigens to protect against the phylogenetically related apicomplexan malaria parasite in non-human primates. It was found that GLA-SE was needed for immunization of primates even when this was not the case in mice. In other earlier work, separate constituents of GLA-SE were used. GLA alone was called “GLA-AF” when it was prepared in an aqueous formulation. This formulation did not contain an emulsion or extra excipients so it is not equivalent to the GLA in GLA-SE. GLA-AF, SE alone and GLA-SE formulations were compared in some of these earlier studies. In almost all of these different systems the value of using both GLA and SE together was proven (Table 1).^20–23, 33–36^ This work has been advanced to the clinic, demonstrating both efficacy and safety in studies with GLA and SE formulated and administered together. We leveraged this extensive, earlier experience to produce our GLA-SE adjuvanted SAPN vaccine for *T. gondii*.

In our work with this new SAPN-design, the flagellin molecule itself is an integral part of the SAPN scaffold (Fig. 1b), with or without the A11 CD8^+^ T cell-eliciting epitopes. This SAPN scaffold lacking the CD8+ epitopes conferred only a small amount of protection compared with the scaffold with the inclusion of the A11 peptides. It is not possible to create a relevant separate control without flagellin because in this scaffold the HLA A11 binding peptides are intercalated into the flagellin molecule itself as shown in Fig. 1.

The location of CD8^+^ epitopes within a protein sequence has been shown to be critical. Our arrangement of peptide epitopes into a polypeptide induced robust immunity.^25^ Deconvolution of peptide components has shown that certain epitopes alone may have different toxicity when separated from other peptides (El Bissati, McLeod, et al., in preparation). We already know that the adjuvanted polypeptide can protect in studies that are described in a separate manuscript.^25^ We also found that the HLA Class 1, A*1101 interacting peptides are specific for HLA A*1101 and not to other HLA supermotifs B7 or A2.^25^ Further, we demonstrated that the mouse C57Bl6 macrophages cannot present these peptides to HLA A*1101 T cells.^25^

Further, these five CD8^+^ epitopes, as well as full-length proteins from which they originate, were characterized to determine how well conserved the proteins, and especially the specific peptides we included, are across multiple strains of genetically divergent parasites from different geographic regions (Tables 2–4. Octamer/nonamer peptides; Supplementary figures S1 [SAG1], S2 [GRA6], S3 [GRA5], S4 [SAG2E], and S5 [SRS52A]). This analysis of 62 genetically divergent strains (Fig. 1a) supports the use of an immunosense approach. This approach creates a single protein which contains the relevant epitopes but does not include extraneous epitopes that are potentially harmful, as certain *T. gondii* epitopes are known to be. There were far fewer polymorphisms in our smaller octamer nonamer peptides (Tables 2–4) than in the full-length proteins (Table 3, Figs. S1–S5). There were good binding octamer/nonamers for three A11 epitopes among all the different genetic isolates (Table 4). The predicted binding scores for the peptides from the many genetically polymorphic strains were high for all but two peptides (Table 4). In contrast, it would take many full-length variants to obtain good geographic coverage of either of these polymorphic proteins if peptides other than our octamer/nonamer were critical to that protection. This provides further conceptual support for using an immunosense approach to make a vaccine with, potential to work well, and most parsimoniously, in many geographic areas. There are recent data concerning the unique processing of *T. gondii* proteins in human cells. Quite remarkably, there are longer (likely decoy) peptides that bind HLA-A2 in the natural infection of THP1 cells.^37^ This makes our targeted immunosense approach, beginning with human cells, then using HLA transgenic mice, especially valuable in creating a vaccine for people, rather than mice. These are critical considerations important beyond a vaccine protective against just this organism, when one wants to have broad utility across different demographics, worldwide. This is what we are working toward because of the substantial disease burden of toxoplasmosis as a global clinical problem.^38^

Data from studies of human cells and mice, in our work, considered together, demonstrate the robust and practical use of this model system.^3–6, 25^ Antigen processing and presentation in humans and mice have differences that are well known. For example, as shown elegantly, recently,^39^ human and murine tapasin diverge in sequence. These tapasins are chaperones of MHC class I molecules. Newer murine models with human tapasin and proteasome might improve vaccine potency and be more relevant to human vaccine development.^39^ Alternatively, our optimized cleavage sites, based on human infection, using human PBMC, may work as effectively in our HLA-A*1101 mice. This comparison will be tested in future studies. Further, there are other significant differences between mice and humans. Murine models have been imperfect predictors of vaccine performance in humans, apart from differences in HLA, tapasin or variants of *ERAP*. Differences in ERAP between mice and humans does not cause differences in processing and presentation. Other differences between mice and humans include very different total blood volume, rate of metabolism, skin composition/absorption rate, different TLR specificities, as well as other basic differences between murine and human cells. For example, human T cells, express Class II MHC, while mouse T cells do not. Nonetheless, relating to the question of whether HLA transgenic mice are a good approximation of human responses, Sette et al. made the following comment: despite the differences, when this issue was considered in detail, practical studies confirm that “although there *ought to be a difference*, actual data in peer reviewed studies, show that *there is little difference”*.^40^ Humanized mice with blood and liver transplants and other modifications are also still imperfect. These humanized mouse systems have not yet worked really well to demonstrate practical feasibility. For vaccine development, the FDA requires animal in vivo immunogenic data for IND submission. Vaccine developers have followed this requirement for the vaccines they are creating, which include viral vector or VLP-based vaccines. To date, there is no system that allows skipping the animal testing step. Most vaccines (e.g. viral, virus-like particles, proteins, bacterial) generate an immune response in an animal model. These immune responses provide preliminary data prior to studies in non-human primates and then in humans. This approach permits one to conclude that the vaccine is safe and ‘active’, even if the animal model is not absolutely predictive of precise human correlates, although that would be ideal. We have found that our approach appears to provide insight and to work effectively.^3–5, 25^ This approach includes using bioinformatics, testing human cells for immunogenicity, and then testing those down-selected peptides re-assembled into a protein with linkers designed for proper cleavage.^41^ This is followed by testing for efficacy and safety using HLA transgenic mice.^3–5, 25^ This approach is shown in our data herein, in our previous foundational experiments,^25^ and also by many others using other systems. This is for both immunogenicity of peptides or polypeptides or DNA or RNA in human cells first. This is then extended to murine cells, followed by protection measured as reduced parasite burden and enhanced survival. Although imperfect, there is considerable prior support, and support in these recent studies. The use of HLA transgenic mice can obviate problems of heterogeneity, both for MHC supermotifs, and parasite isolates. This is in a proven practical manner in vaccine development.^19, 37, 42–52^ The issues of genetic polymorphism, as well as potential harmful constituents, are amplified in full-length natural proteins (Tables 1–4; Fig. 1, S1–S5). Thus, the choice of peptides that are sufficient to interact with HLA molecules that are present in more than 90% of the human population can be made using bioinformatics in a rational and parsimonious manner. This approach considers parasite genetic variation in an inductive, immunosense manner that is proving to be valuable for development of vaccines for humans.

The inclusion of flagellin into immunogens can serve as a potential adjuvant.^53-57^ There is experience where flagellin has been safe and effective as an adjuvant in pre-clinical animal studies.^53–57^ It also has been effective when used for immunizations of both younger and older persons in clinical trials for influenza vaccines.^53–57^ There is a robust literature which describes studies of the mechanisms whereby this TLR5 ligand functions as an adjuvant.^53-57^

**Fig. 6:**
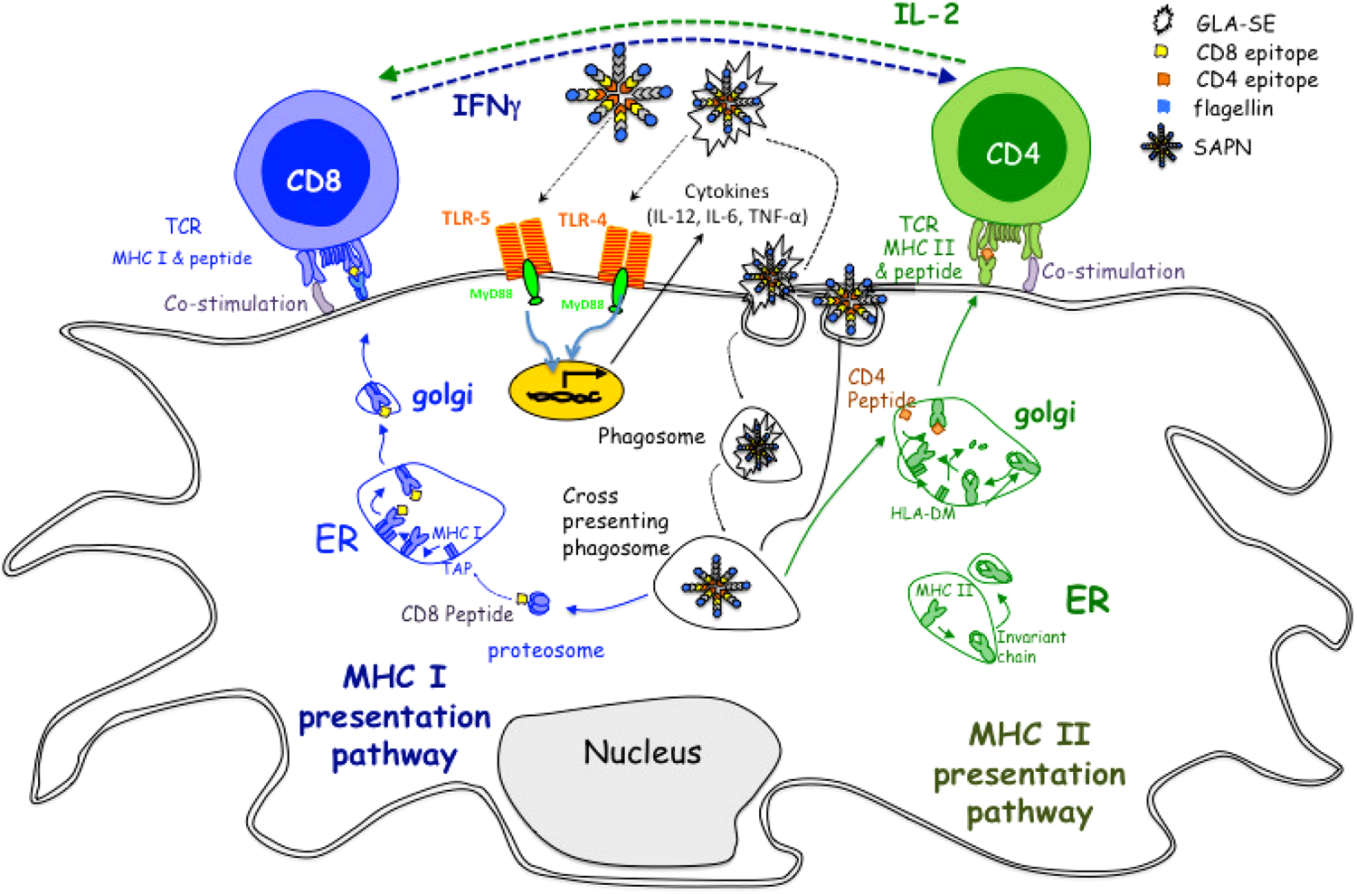
SAPN adjuvanted with GLA-SE have peptides that are presented by MHC molecules on the follicular dendritic cells^14^ to T lymphocytes. GLA-SE and flagellin are ligands of TLR-4 and TLR-5 receptors, respectively. Ligating these receptors leads to the production of proinflammatory cytokines (IL-12, IL-6, TNF-α) and the expression of co-stimulatory molecules on the antigen-presenting cell surface. It remains to be determined whether the GLA-SE emulsion independently ligates TLR4 or whether SAPN are entrapped in the emulsion when this occurs, so both possibilities are shown. Original diagram for polyepitope for 5 A11 peptides^25^ provide a foundation to which concepts demonstrated in studies herein were added

In summary, our study showed that a SAPN-protein chain with five CD8^+^ T cell-eliciting MHC class I epitopes from *T. gondii*, and the MHC class II epitope PADRE, can be refolded to form a nanoparticle. Using HLA-A*1101 transgenic mice, we demonstrate that the SAPN emulsified in GLA-SE adjuvant elicits a protective MHC class I response. Thus, our work demonstrates that we have developed an improved assembly of peptides for cross presentation of CD8^+^ T cell eliciting epitopes (Fig. 6) in vaccines to prevent toxoplasmosis.

## Materials and methods

### Peptides

KSFKDILPK (SAG1_224–232_), STFWPCLLR (SAG2C_13–21_), AVVSLLRLLK (GRA5_89–98_), SSAYVFSVK (SRS52A_250–258_), AMLTAFFLR (GRA6_164–172_)^25^ and PADRE, a universal CD4^+^ helper epitope (AKFVAAWTLKAAA)^26^ were used in the vaccine constructs.^25, 52^ Infectious Diseases Research Institute (Seattle, Washington) synthesized the TLR4 agonist adjuvant called GLA-SE.^3–6, 20–23, 25^ This was prepared and used as a stable oil-in-water emulsion.

### Molecular biology

The methods using DNA coding for the nanoparticle constructs were similar to those described in our earlier work.^18^ Briefly, they were prepared using standard molecular biology procedures as described in our earlier work from our laboratory by Babapoor et al.^18^ Specifically, plasmids containing the DNA coding for the protein sequence were used.^18^ They were constructed by cloning into restriction sites in the SAPN expression plasmid.^18^ We used a SAPN construct we had developed and described earlier.^18^ Briefly, this construct is composed of a pentameric coiled-coil tryptophan zipper.^18^ This zipper is linked by a glycine residue to a trimeric de-novo designed leucine zipper coiled coil.^18^ In this construct, a flagellin construct composed of the D0 and D1 domains (residues 1–177 and 249–372) of *Salmonella enterica* flagellin from the structure with pdb-code 3V47 from the RCSB protein data bank is used to extend the protein chain at the C-terminus^18^ (Fig. 1).

The CD8^+^-peptide sequence AVVSLLRLLKNAMLTAFFLRNAAAKSFKDILPKKAAASSAYVFSVKKAAAKFVAAWTLKAAAKSTFWPCLLR with the five CD8^+^ epitopes^25^ also containing PADRE^25, 26^ was next inserted into the D1 domain of flagellin.^18^ This polypeptide completely replaces the D2 and D3 domains to generate the CD8^+^ T cell-eliciting SAPN called “***CD8-SAPN***”.^18^ Overall, the positive charge of this epitope string is balanced with stretches of negative charges at both ends of the epitope sequence.^18^ Our *Empty-SAPN* was generated using the short linker KYKDGKGDDK to replace the D2 and D3 domains of flagellin.

### Protein expression

This was performed exactly as we had performed and described in our earlier work from our laboratory by Babapoor et al.:^18^ Plasmids were transformed into *Escherichia coli* BL21 (DE3) cells.^18^ *E. coli* were grown at 37 °C in Luria broth with ampicillin.^18^ We induced expression using isopropyl β-D-thiogalacto-pyranoside. Cells were removed from 37 °C 4 h after induction.^18^ They were harvested by centrifugation at 4000 x g. We stored the cell pellet at −80 °C. We then thawed the cell pellet, keeping it on ice.^18^ We then suspended the pellet in a lysis buffer consisting of 9 M urea, 100 mM NaH_2_PO_4_, 10 mM Tris pH 8, 20 mM imidazole, and 0.2 mM Tris-2-carboxyethyl phosphine (TCEP). SDS-PAGE was used to assess our protein expression level.^18^

### Protein purification

The same methodology we used earlier was used.^18^ Briefly, sonication was used to lyse cells, as described from our laboratory earlier.^18^ Centrifugation at 30,500 × g for 45 min^18^ was used to clarify the lysate. Then, for at least 1 h, our cleared lysate was incubated with Ni-NTA Agarose Beads (Qiagen, Valencia, CA, USA). Next, the column was washed with lysis buffer. This was followed by a wash with a buffer containing 9 M urea, 500 mM NaH_2_PO_4_, 10 mM Tris pH 8, 20 mM imidazole, and 0.2 mM TCEP. A pH gradient was used to purify the protein while bound to the column. The pH gradient for these wash steps was created as follows: 9 M urea, 100 mM NaH_2_PO_4_, 20 mM citrate, 20 mM imidazole, and 0.2 mM TCEP,^18^ with subsequent washes performed at pH 6.3, 5.9, and 4.5.^18^ To elute the protein, we used the lysis buffer, after the pH gradient, with a gradient of increasing imidazole concentrations.^18^

### Protein refolding

We used methodology we have described in our earlier work.^18^ Specifically, for refolding, our protein was first rebuffered to the following conditions: 9 M urea, 20 mM Tris pH 8.5, 50 mM NaCl, 5% glycerol, 2 mM EDTA.^18^ 4 µl of a solution with a concentration of 1.8 mg/ml protein was added to the same buffer solution without urea to a final concentration of 0.05 mg/ml for quick refolding of a first screen.^18^ We used this because this quick dilution from denaturing (urea) to native (no urea) buffer conditions triggers refolding of protein.^18^ We then used negative stain transmission electron microscopy at different resolutions to analyze our solution.^18^ Next, we used further screens for optimal refolding conditions.^18^ These were performed with smaller sampling sizes of the pH and ionic strength.^18^

### In vitro TLR5 response assay

The methods were the same as those used in our recent work.^28^ Activation through TLR5 was assessed for SAPN as we described recently.^24^ Testing was done using TLR/NF-κB/SEAPorter™ Stably Transfected HEK 293 Cell Lines (Novus Biologicals, Littleton, CO; tested for *Mycoplasma* but not authenticated by STR profiling) as follows: All cell lines were stably co-transfected cell lines which express TLR5 and have a secreted alkaline phosphatase (SEAP) reporter gene under transcriptional control of an NF-κB response element. Fourteen thousand cells per well were seeded in a 96-well plate at passages 5–9. 20-4 h later, we removed growth media. Growth media was replaced with DMEM high glucose (Hyclone, Logan, UT). This contained either a SAPN, or recombinant flagellin (Novus Biologicals), at concentrations of 0.1, 1, 10, 100, 1000 ng/mL, each in triplicate. Media alone was present in control wells. Wells were exposed to the stimulus for 24 h. Then, supernatant was collected and used to determine whether SEAP was present. This was determined with a Reporter Assay kit for SEAP (Novus Biologicals). This was done using the manufacturer’s instructions. Media- only controls were used to normalize SEAP activity. This was used to determine each construct’s EC50. Triplicate determinations were utilized for each experimental condition.

### Mice

The mice were those we created and described earlier.^5, 23^ The methods were identical to those used in our earlier work.^3–6, 23^ Specifically, “HLA-A*1101/K^b^ transgenic female mice were generated and then bred/produced at Pharmexa-Epimmune (San Diego, CA).^23^ They were then embryo-rederived at Taconic and JAX laboratories.^23^ Colonies were then expanded and they were then maintained and produced in isolators at the University of Chicago.^23^ These mice express a chimeric gene called HLA-A*1101/K^b^ transgene.^23^ This chimeric gene consists of the 1st and 2nd domains of HLA-A*1101 and the 3rd domain of H-2K^b^.^23^ Mice were between 10 and 14 weeks of age in experiments. Mice were maintained in SPF conditions throughout.^23^ All of our studies were performed with the Institutional Animal Care and Use Committee at the University of Chicago’s review, approval, and oversight.

### Immunizations of mice and challenge

To assess the immunogenicity of the SAPNs, mice with the HLA-A*1101 transgene were inoculated intramusculary. In this injection, 20 μg SAPN was emulsified in the TLR4 agonist, i.e., 5 μg of GLA-SE. The immunizations were administered three times at 2 weeks intervals. For the experiments in which these mice were challenged, challenge was at 14 days post-immunization.^3^ Specifically, they were challenged intraperitoneally using 2000 Type II (Me49-Fluc) parasites.^23^

### ELISpot assay to determine murine splenocyte immune responses

This was performed as described in our earlier work which provided the foundation for our own present studies.^3–6, 25^ Specifically, spleens were harvested 14 days after immunization^3–6, 25^ as follows: initially, they were pressed through a 70 µm screen.^3–6, 25^ This allowed for formation of a suspension of single-cells. Erythrocytes were depleted from this suspension. AKC lysis buffer (160 mM NH_4_Cl, 10 mM KHCO_3_, 100 mM EDTA) was used to deplete the RBCs.^3–6, 25^ Hank’s Balanced Salt Solution (HBSS) was used to wash splenocytes twice.^3–6, 25^ Then the splenocytes were resuspended in RPMI-1640 supplemented with 2 mM L-GlutaMax.^3–6, 25^ Murine splenocyte ELISPOT assays were performed as described earlier.^3–6, 25^ This was done using anti-mouse IFN-γ mAb (AN18) and biotinylated anti-mouse IFN-γ mAb (R4–6A2).^3–6, 25^ In each well, 2.5–5 × 10^5^ splenocytes were plated.^3–6, 25^ Mabtech (Cincinnati, OH) was the source of all of the antibodies and all of the reagents used to perform ELISPOT assays.^3–6, 25^ A minimum of three replicate wells were used to plate cells for each condition,^3–6, 25^ as described earlier, to measure spot-forming cells per 10^6^ murine splenocytes.^3–6, 25^

### Bioluminescence imaging to determinine outcomes of type II parasite challenge

We imaged mice infected with 2000 Fluc tachyzoites of the Me49 strain of *T. gondii* as described in our earlier work.^25^ Twenty-one days after challenge, an in vivo imaging system (IVIS; Xenogen, Alameda, CA)^25^ allowed us to visualize luciferin injected retroorbitally interacting with luciferase in the parasites.^25^ These mice were anesthetized. Anesthesia was performed in an O_2_-rich induction chamber with 2% isoflurane.^25^ Imaging took place 12 min after receiving luciferin.^25^ Living Image^®^ 2.20.1 software (Xenogen) was used for assessment of photonic emissions.^25^ Pseudocolor representations of light intensity and mean photons/region of interest represent parasite burden in the imaging.^25^ All these mouse experiments were replicated a minimum of two times, as in our earlier work.^25^ In each group we used five mice.

### Enumeration of cysts in mouse brains after type II parasite challenge

Mouse brains were collected at day 21, homogenized in 1 ml of saline (0.85% NaCl), and 50 µl of the homogenate was used to count the tissue cysts, microscopically, as described earlier.^25^ Cyst count was then multiplied by 20. This product then was used to determine the number of tissue cysts per brain.

### Statistical analyses and additional detail concerning animal models

Data were compared for each assay by ANOVA and a Student’s *t*-test. GraphPad Prism 5 software (GraphPad Software, San Diego, CA) was used as described.^6^ ANOVA and multiple comparison procedures identified differences between the groups, as we previously described.^6^ Means ± SD are used to express data. A *p* value <0.05 was considered to be statistically significant for our results.^6^ Sample size in the in vivo studies was selected to be able to detect significant differences in luminescence based on our prior studies.^25^ With 5 per group, there is 80% power to detect a 2-standard deviation difference between groups. With 3 per group, there is 80% power to detect a 2.7-standard deviation difference between groups. All female mice we bred were utilized. They were randomly selected for the different groups but age-matched in the different groups within the experiment. There was no blinding in this experiment. In all in vivo experiments, there were 5 mice per group. In all in vitro experiments, there were 3 mice per group that provided splenocytes. All experiments were replicated at least twice. Representative experiments, of at least 2 separate trials, are shown. There was no data excluded from analyses.

### Data availability

The data that support the findings of this study are available from ToxoDB (http://toxodb.org/toxo/) and the corresponding author on reasonable request.

## Electronic supplementary material

Supplement
