# Supplementary material for: Protein nanovaccine confers robust immunity against Toxoplasma
Source: NPJ Vaccines. 2017 Sep 5;2:24. doi: 10.1038/s41541-017-0024-6 (PMC5627305; doi:10.1038/s41541-017-0024-6)

**Supplement**

# Protein nanovaccine confers robust immunity against *Toxoplasma*

Kamal El Bissati^a*^, Ying Zhou^a^, Sara Maria Paulillo^b^, Senthil Kumar Raman^b^, Christopher P. Karch^c^, Craig W. Roberts^d^, David E. Lanar^e^, Steve Reed^f^, Chris Fox^f^, Darrick Carter^f^, Jeff Alexander^g^, Alessandro Sette^h^, John Sidney^h^, Hernan Lorenzi^i^, Ian J. Begeman^a^, Peter Burkhard^b,c^, Rima McLeod^a,j*^

^a^ Departments of OVS and ^j^ Pediatrics (Infectious Diseases), The University of Chicago, 5841 S Maryland Ave, Chicago, IL 60637, USA.

^b^ Alpha-O Peptides AG, Lörracherstrasse 50, 4125 Riehen, Switzerland.

^c^ Institute of Materials Science and Department of Molecular and Cell Biology, University of Connecticut, 97 North Eagleville Road, Storrs, CT 06269, USA.

^d^ Strathclyde Institute of Pharmacy and Biomedical Sciences, University of Strathclyde, Glasgow, United Kingdom, G4 0RE.

^e^ Walter Reed Army Institute of Research, 503 Robert Grant Ave, Silver Spring, MD 20910

^f^ Infectious Diseases Research Institute, 1616 Eastlake Ave E #400, Seattle, WA 98102

^g^ PaxVax, 3985-A Sorrento Valley Blvd. San Diego, CA 92121

^h^ La Jolla Institute of Allergy and Immunology, 9420 Athena Cir, La Jolla, CA 92037, USA

^i^ J. Craig Venter Institute, J. Craig Venter Institute, 9714 Medical Center Drive,

Rockville, MD 20850

* Corresponding authors. Tel.: +1 773 834 4120. Fax: +1 773 834 3577. E-mail addresses: [rmcleod@bsd.uchicago.edu](mailto:rmcleod@bsd.uchicago.edu), [kelbissati@uchicago.edu](mailto:kelbissati@uchicago.edu)

Classification: Biological Sciences, Medical Sciences.

**Supplement Index**

Figure S1. Multisequence SAG1 alignment

Figure S2. Multisequence GRA6 alignment

Figure S3. Multisequence GRA5 alignment

Figure S4. Multisequence SAG2C alignment

Figure S5. Multisequence SRS52A alignment

**Figure S1**


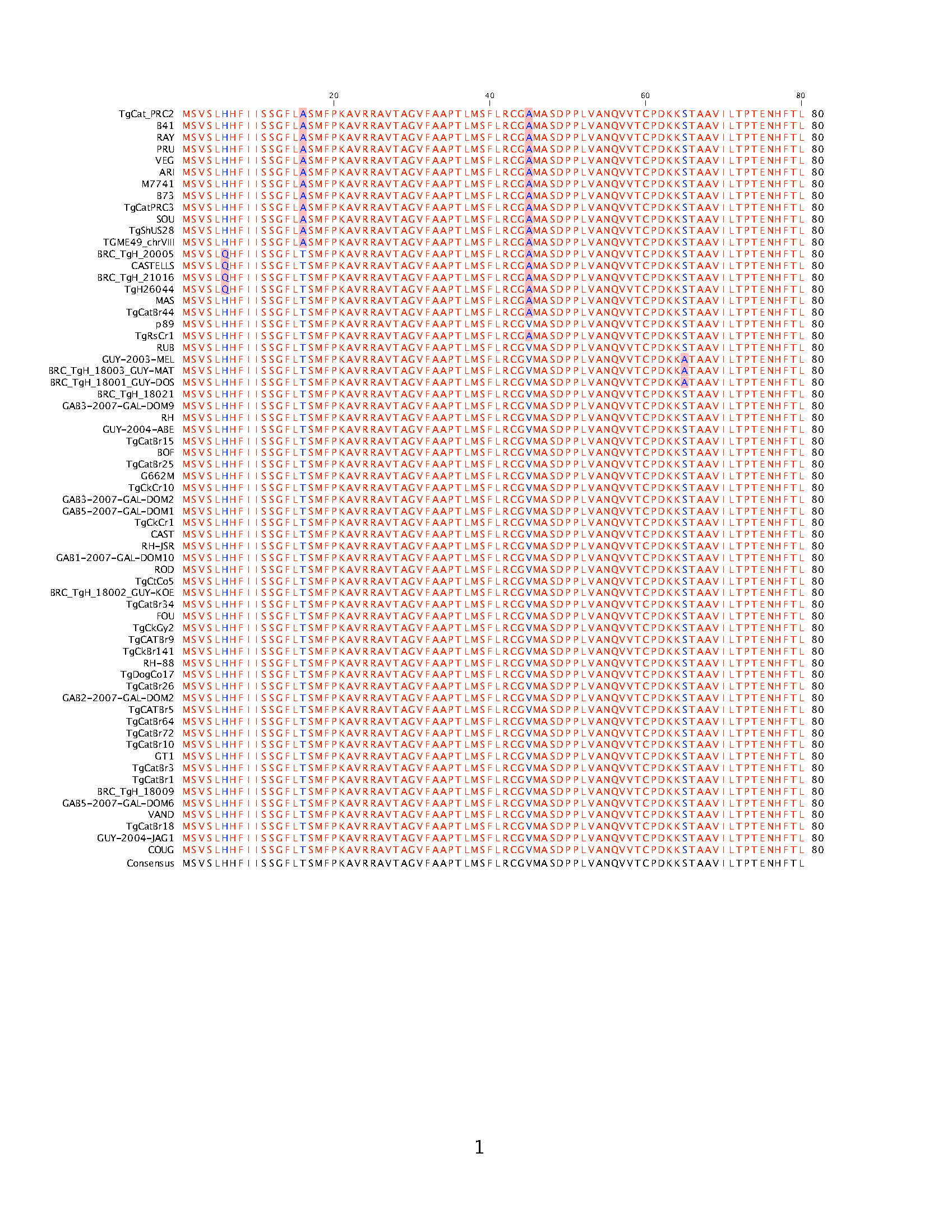


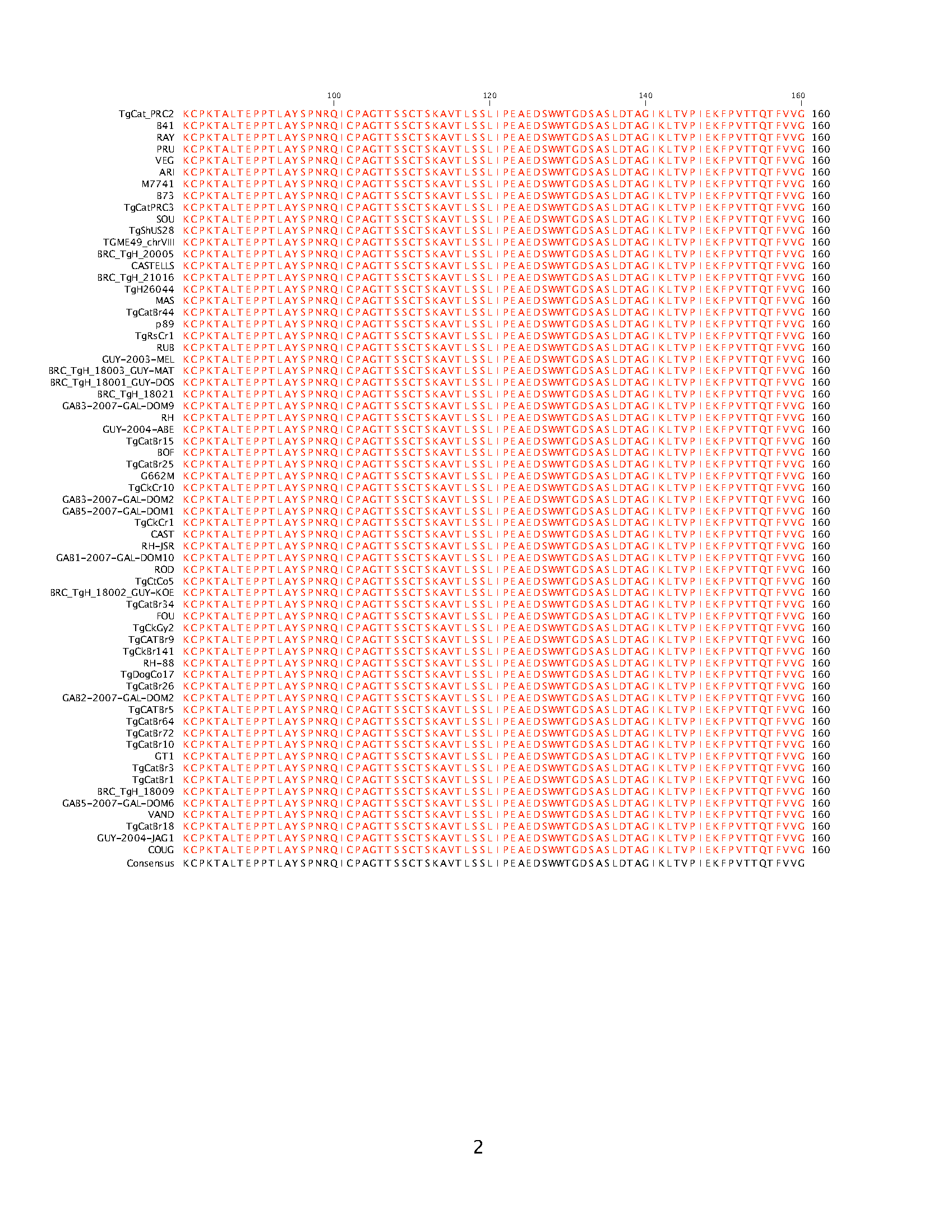


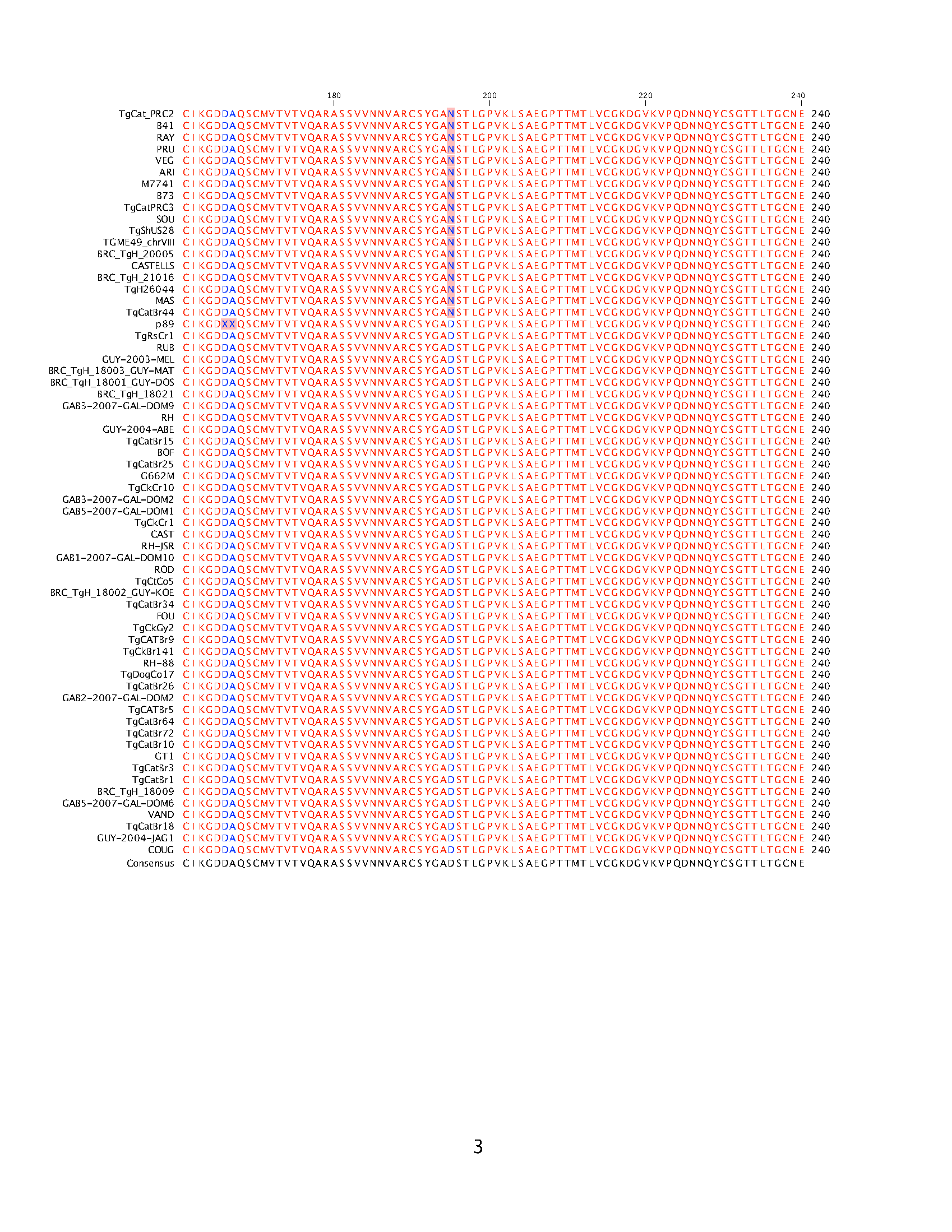


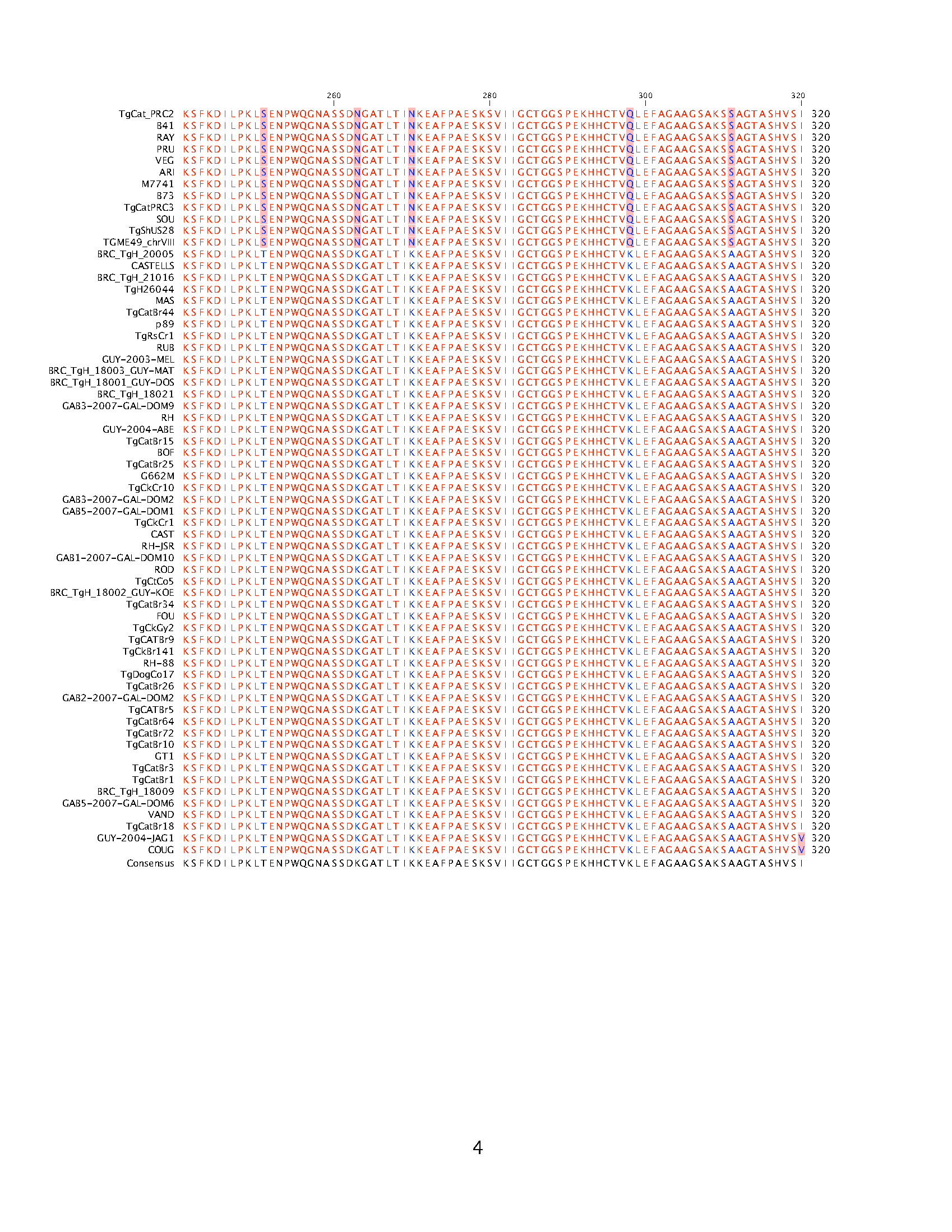


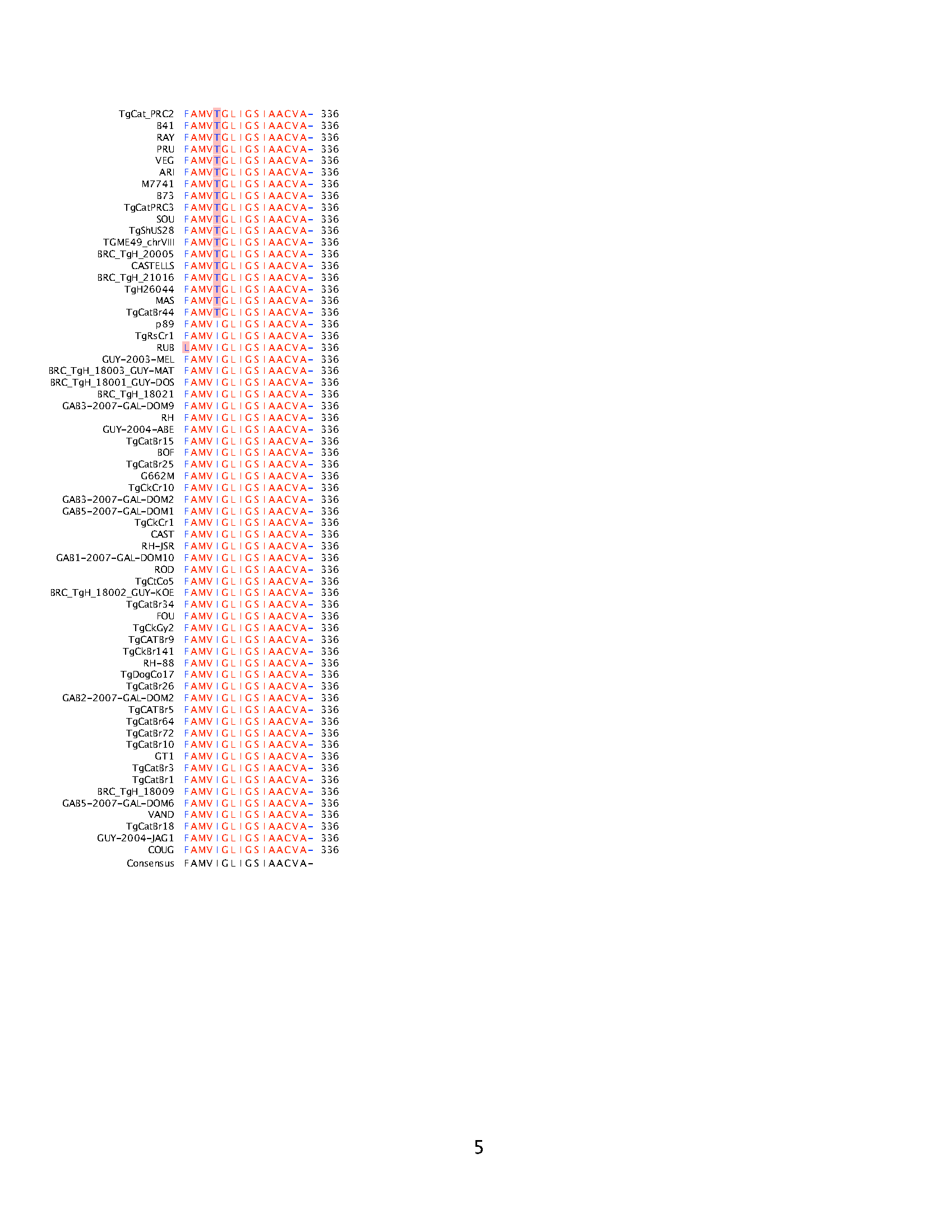


**Figure S2**


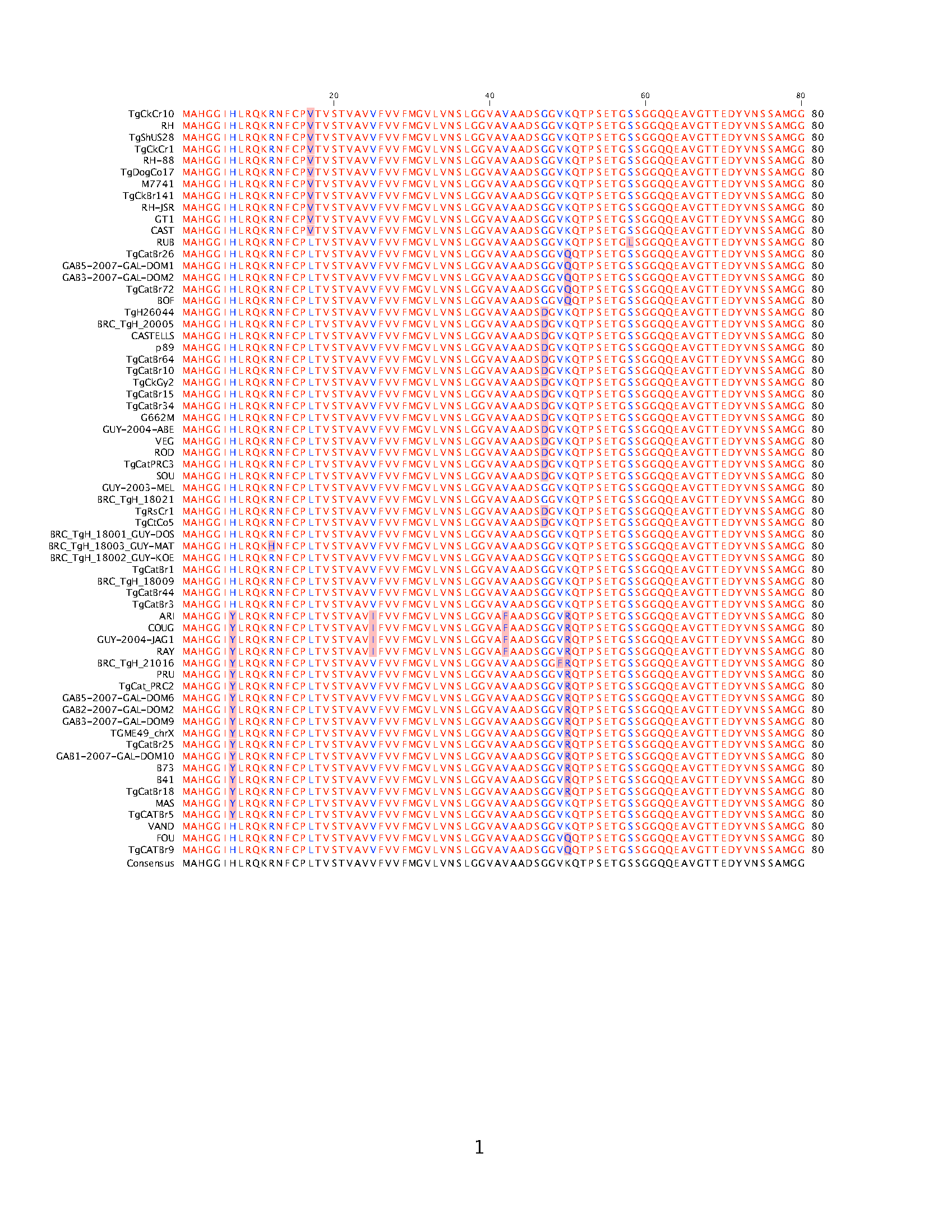


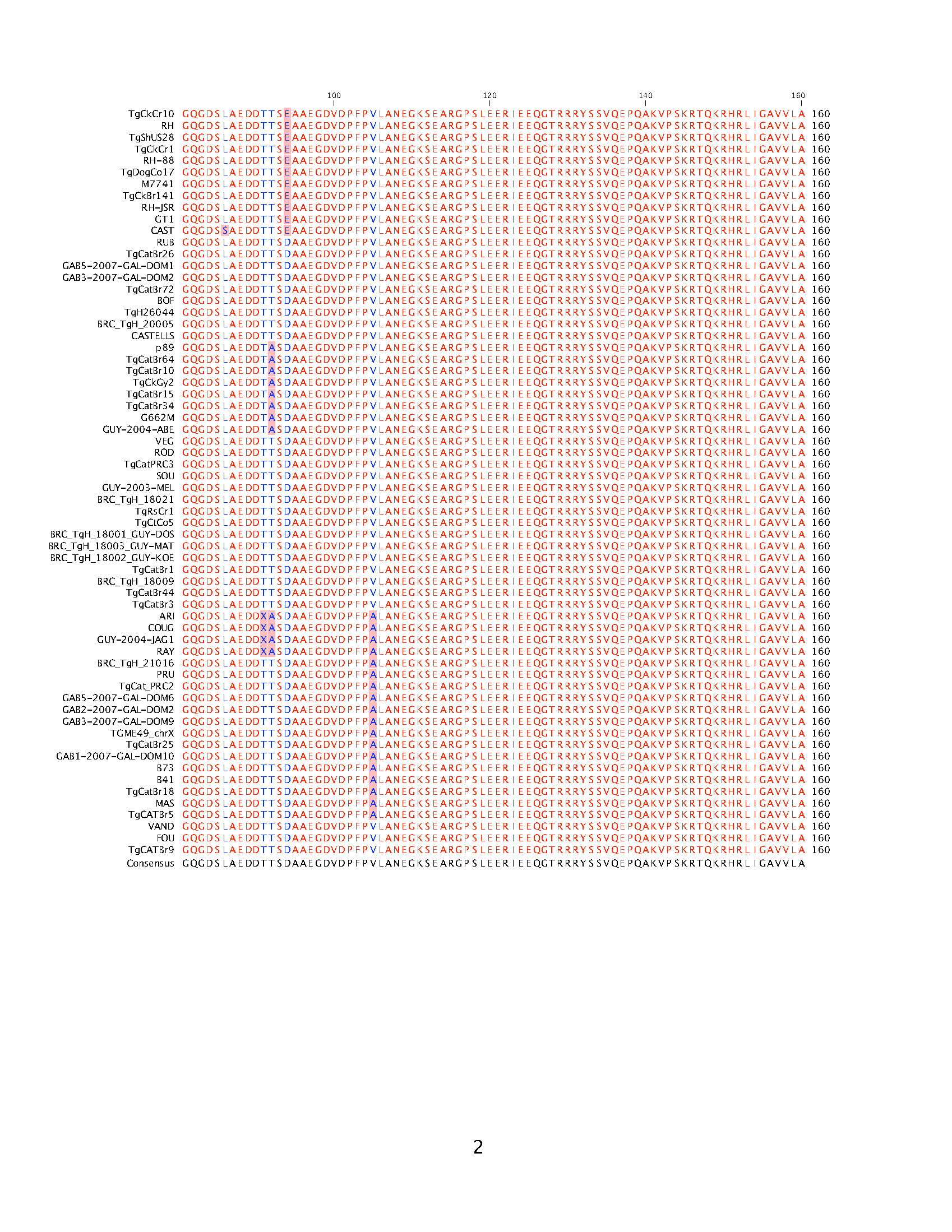


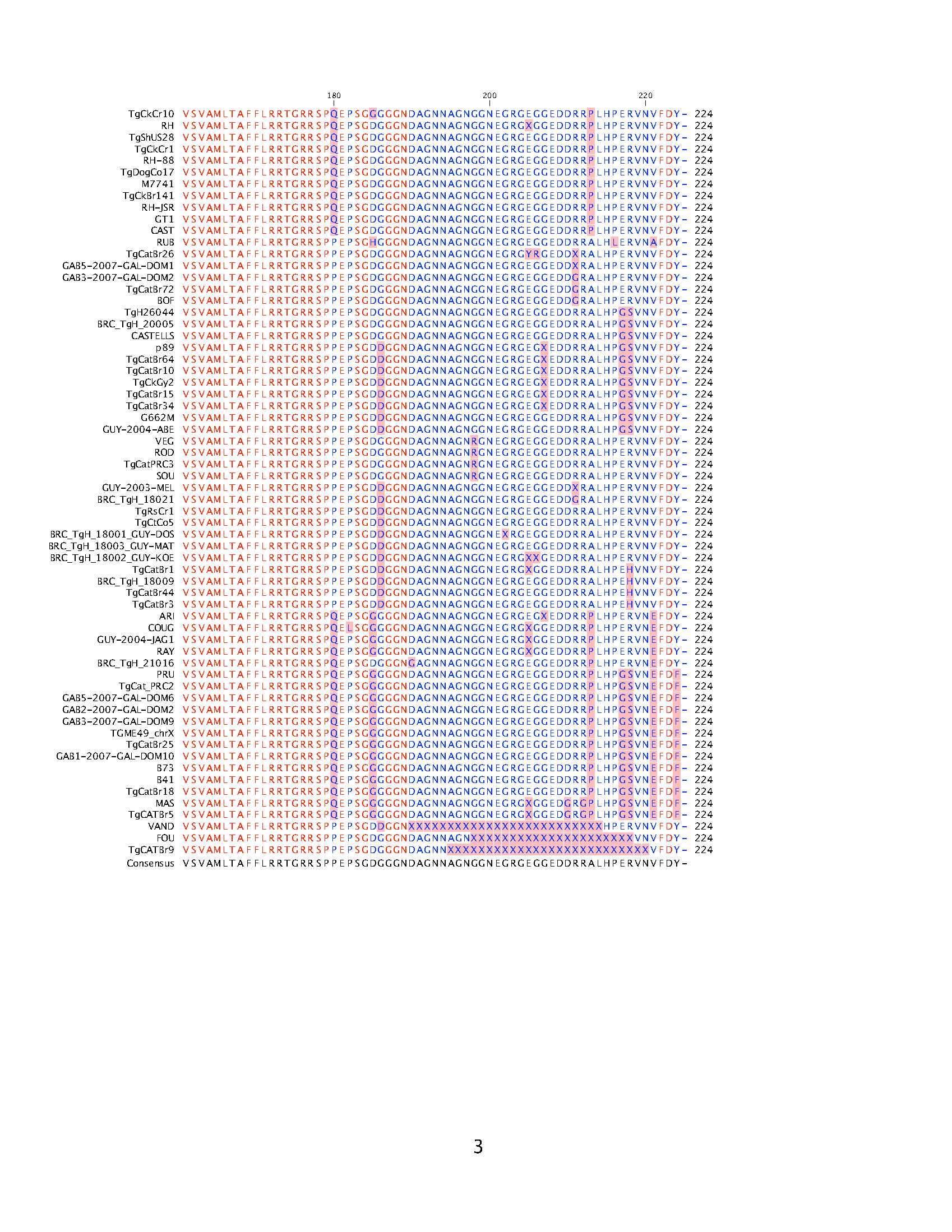


**Figure S3**


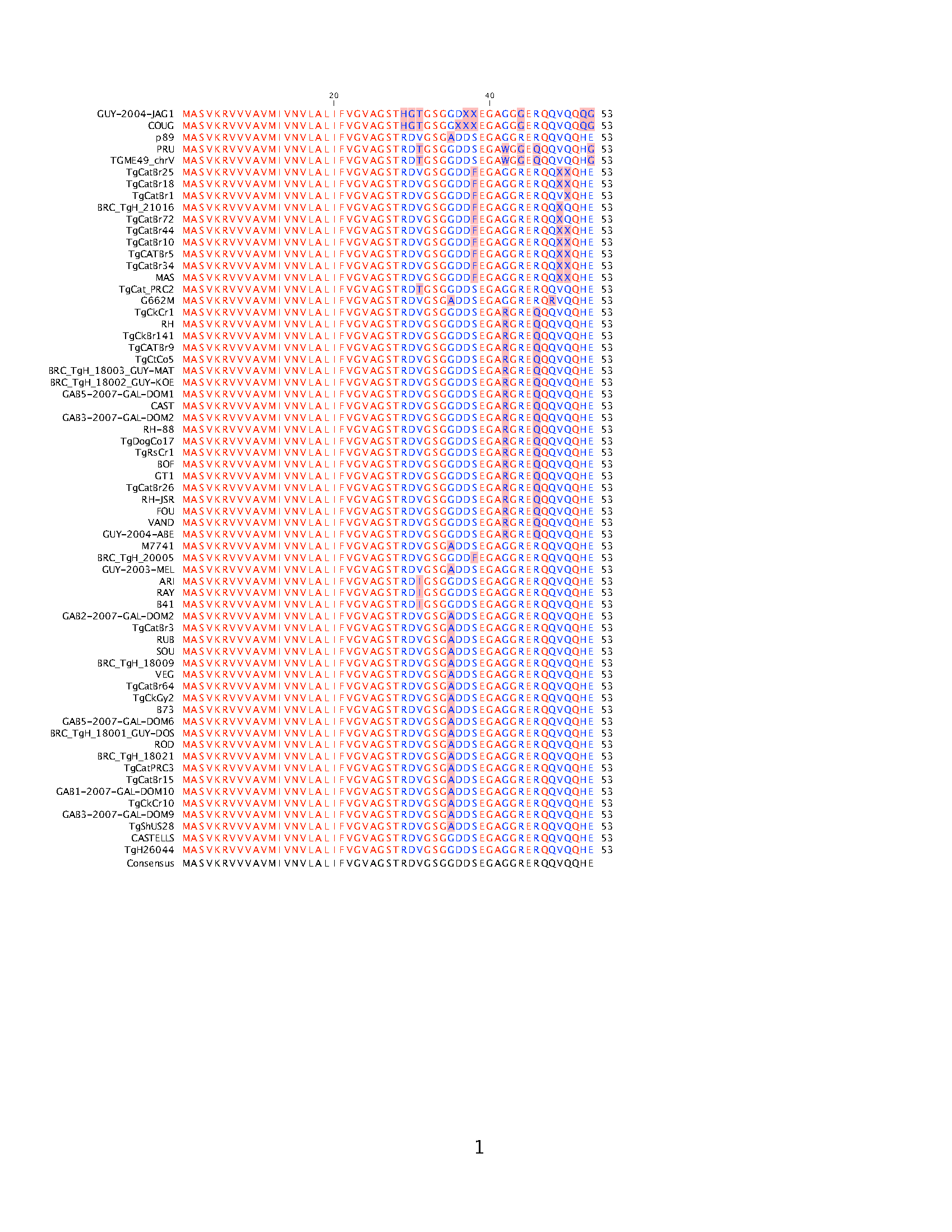


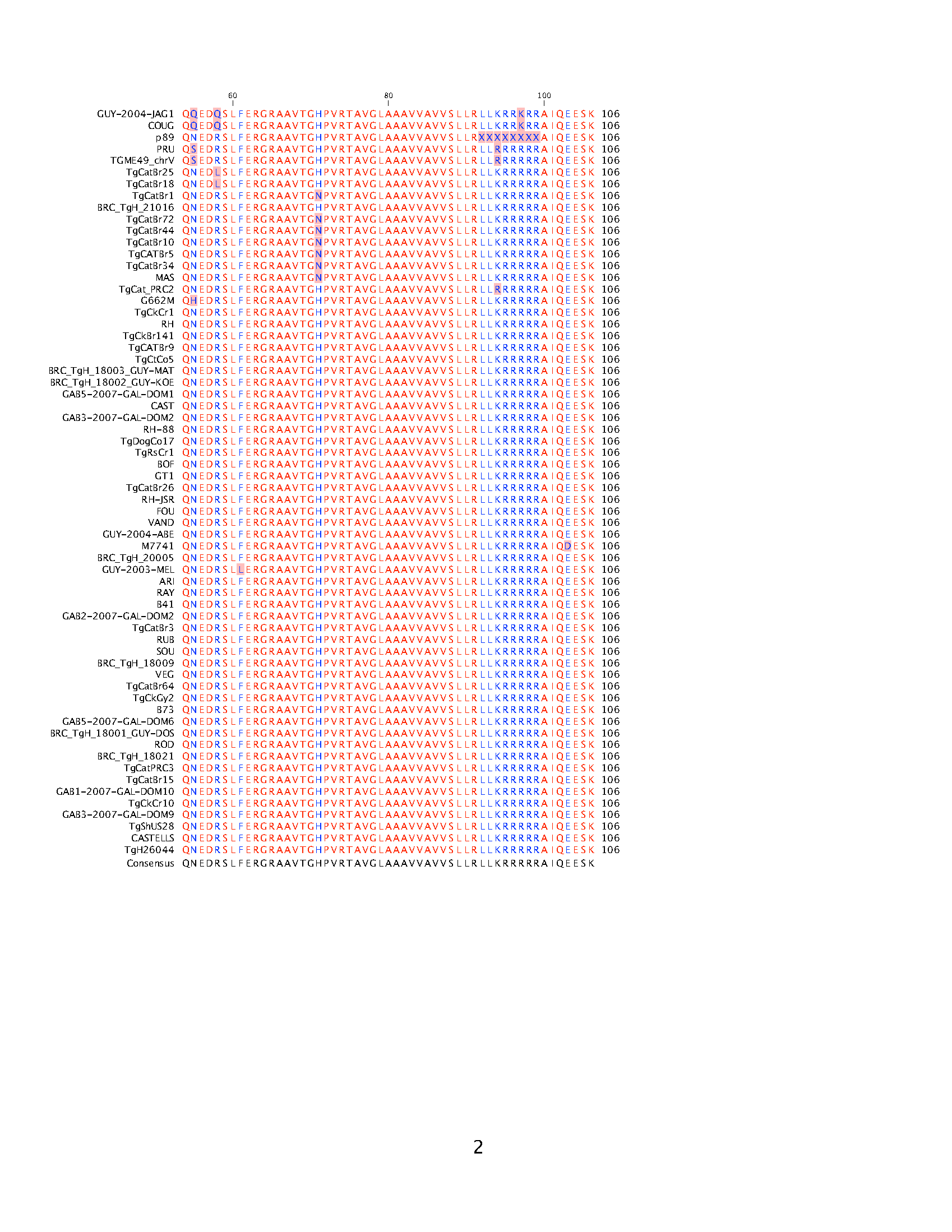


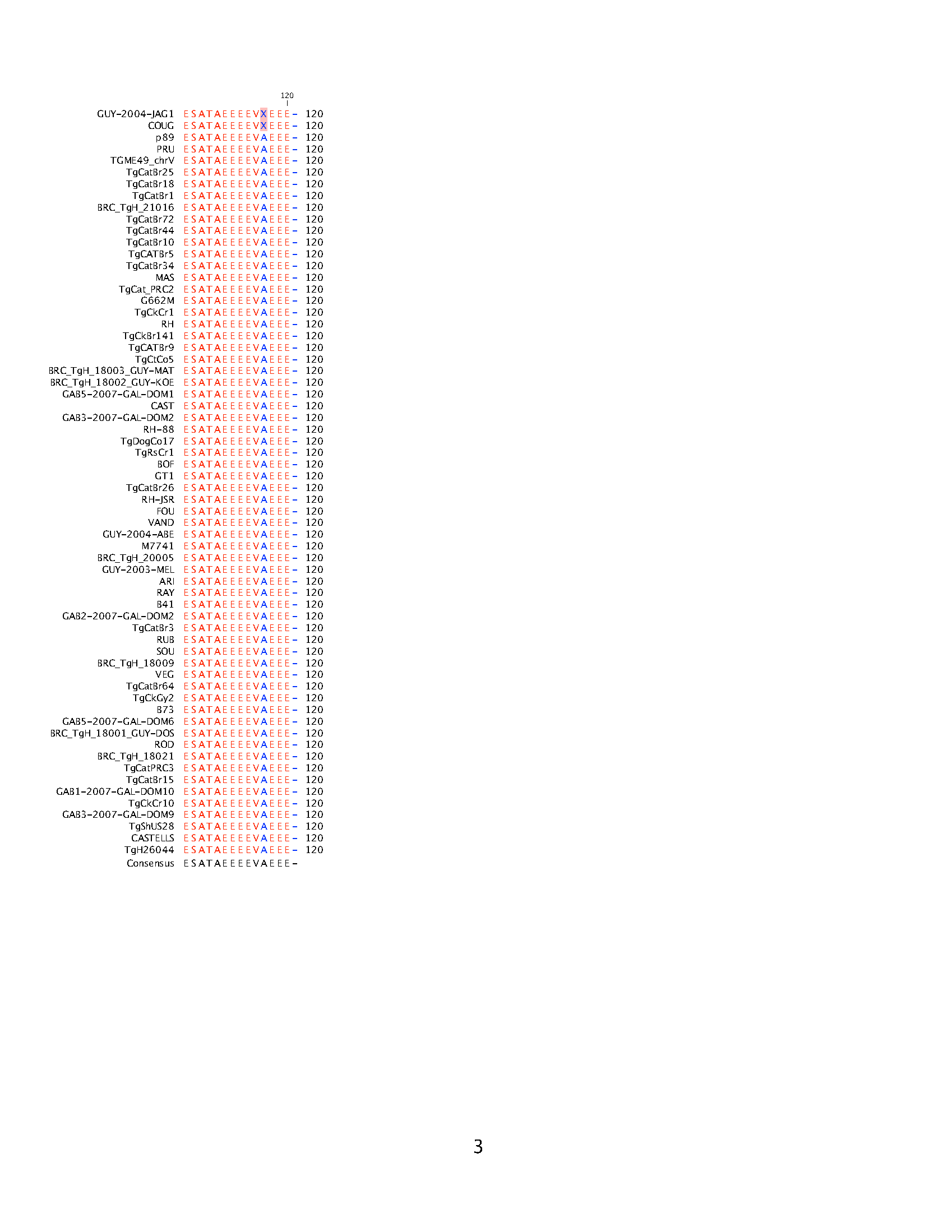


**Figure S4**


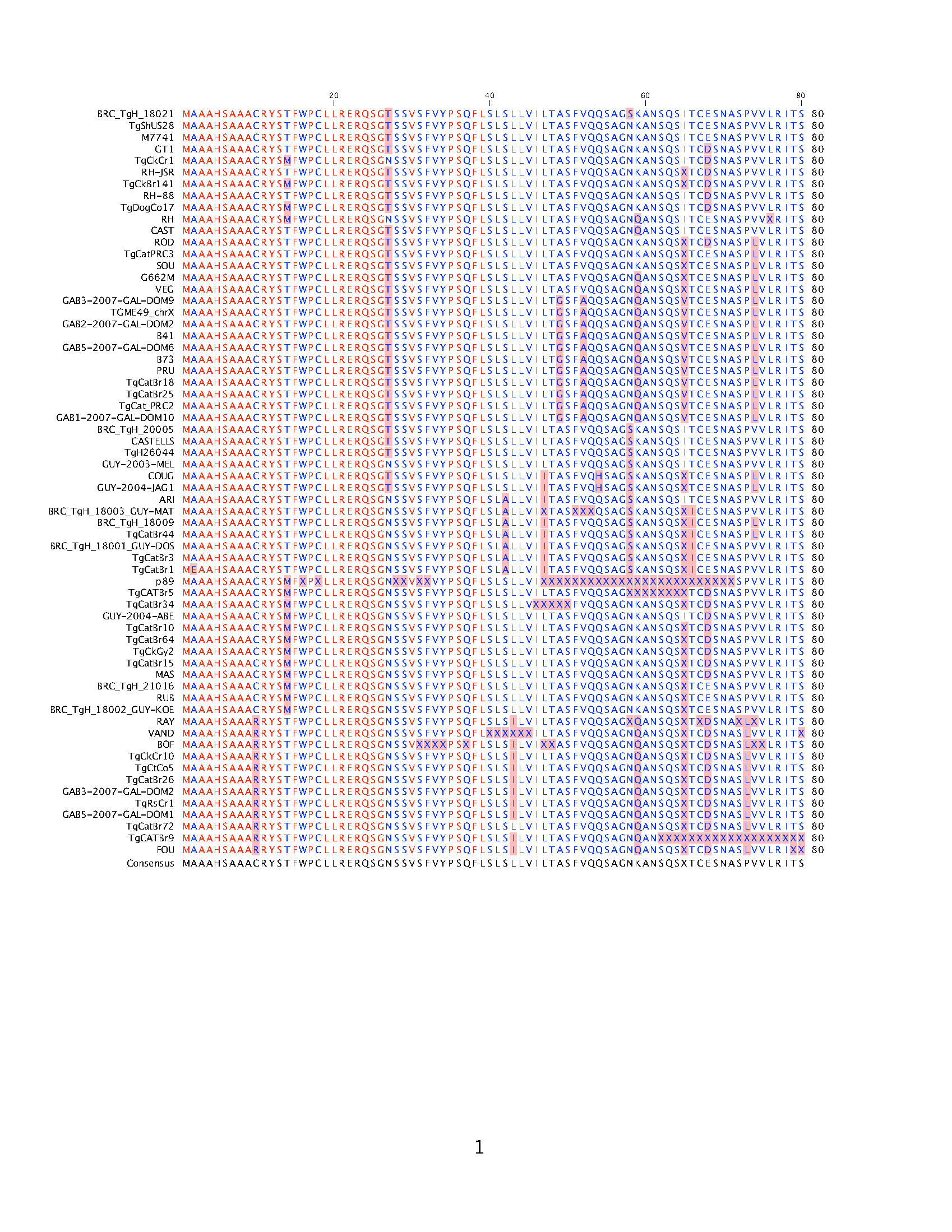


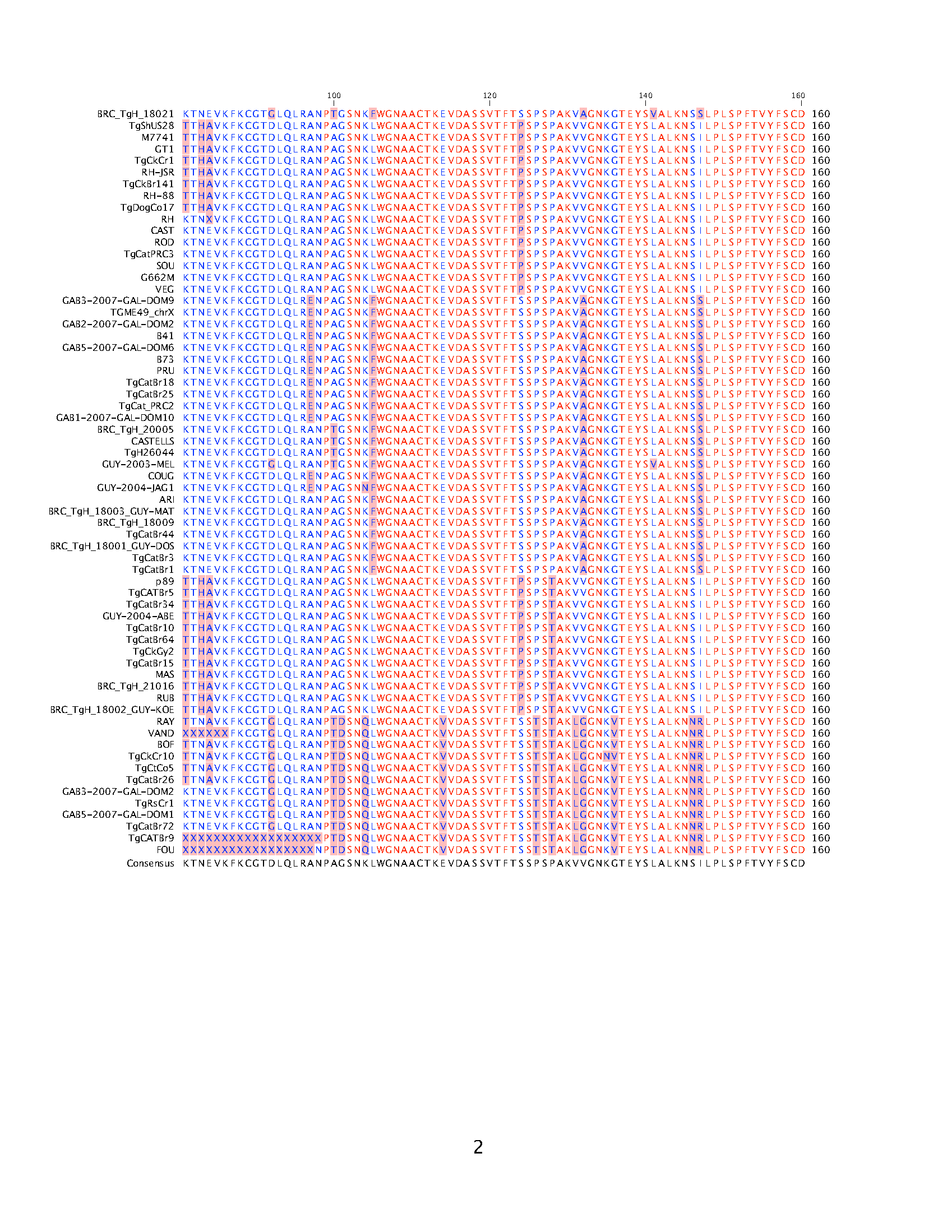


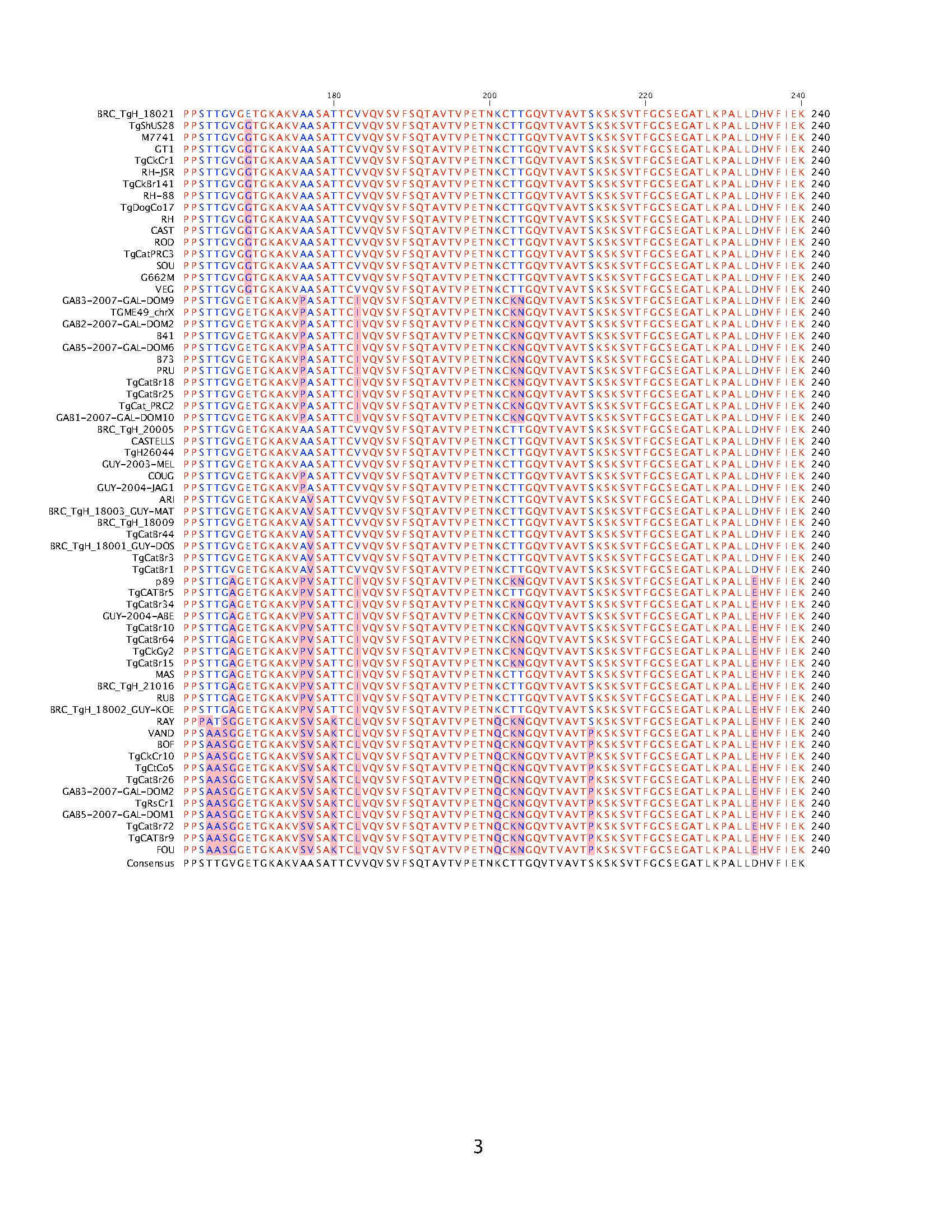


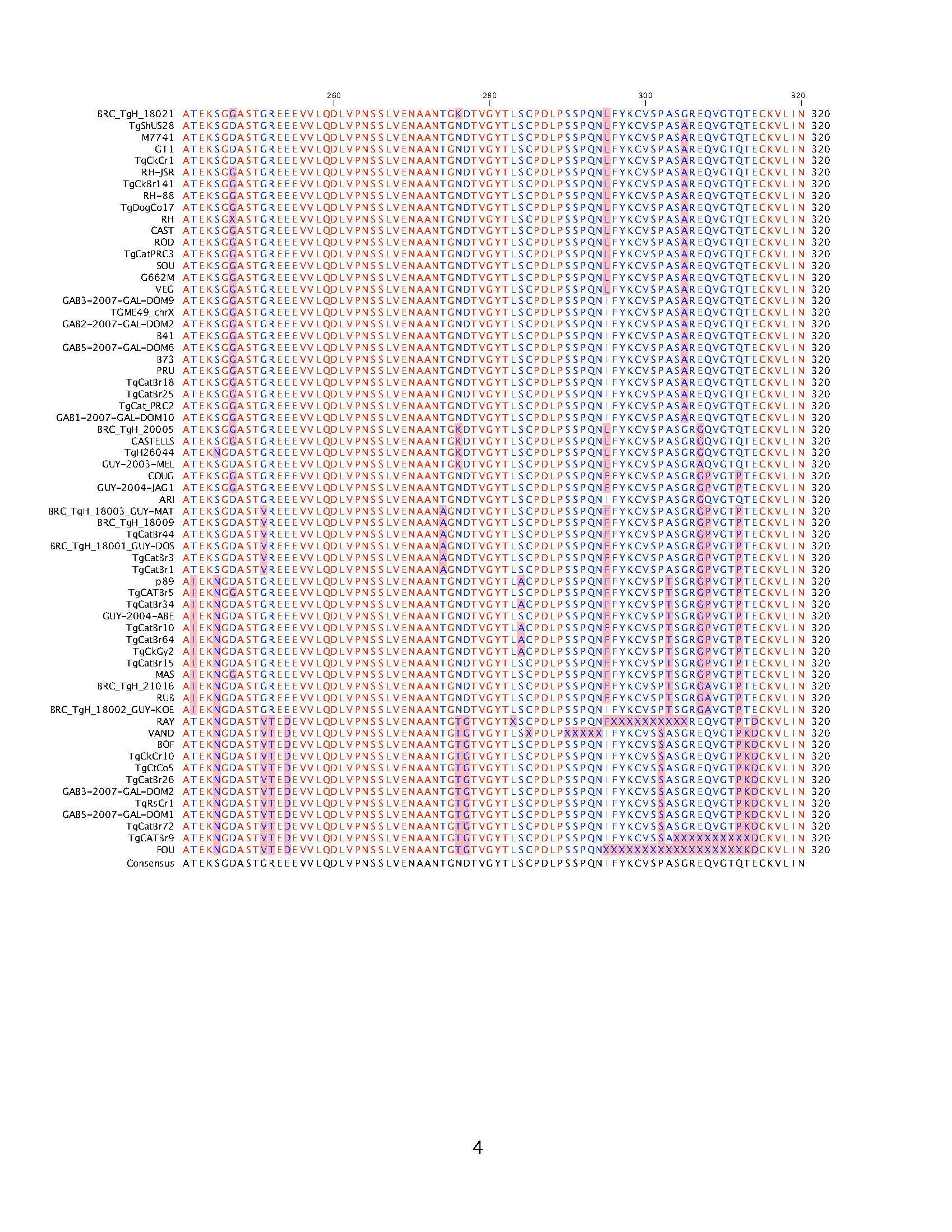


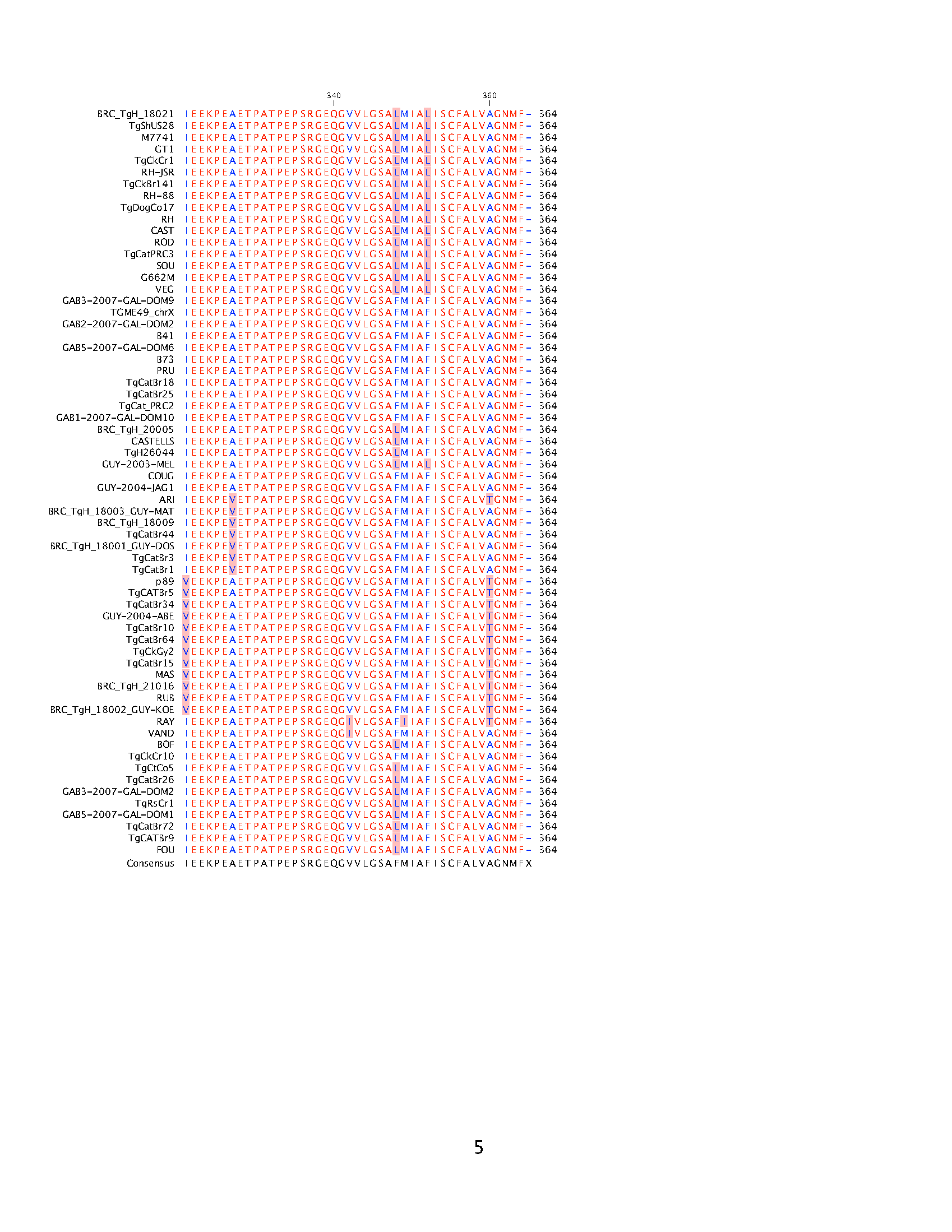


**Figure S5**


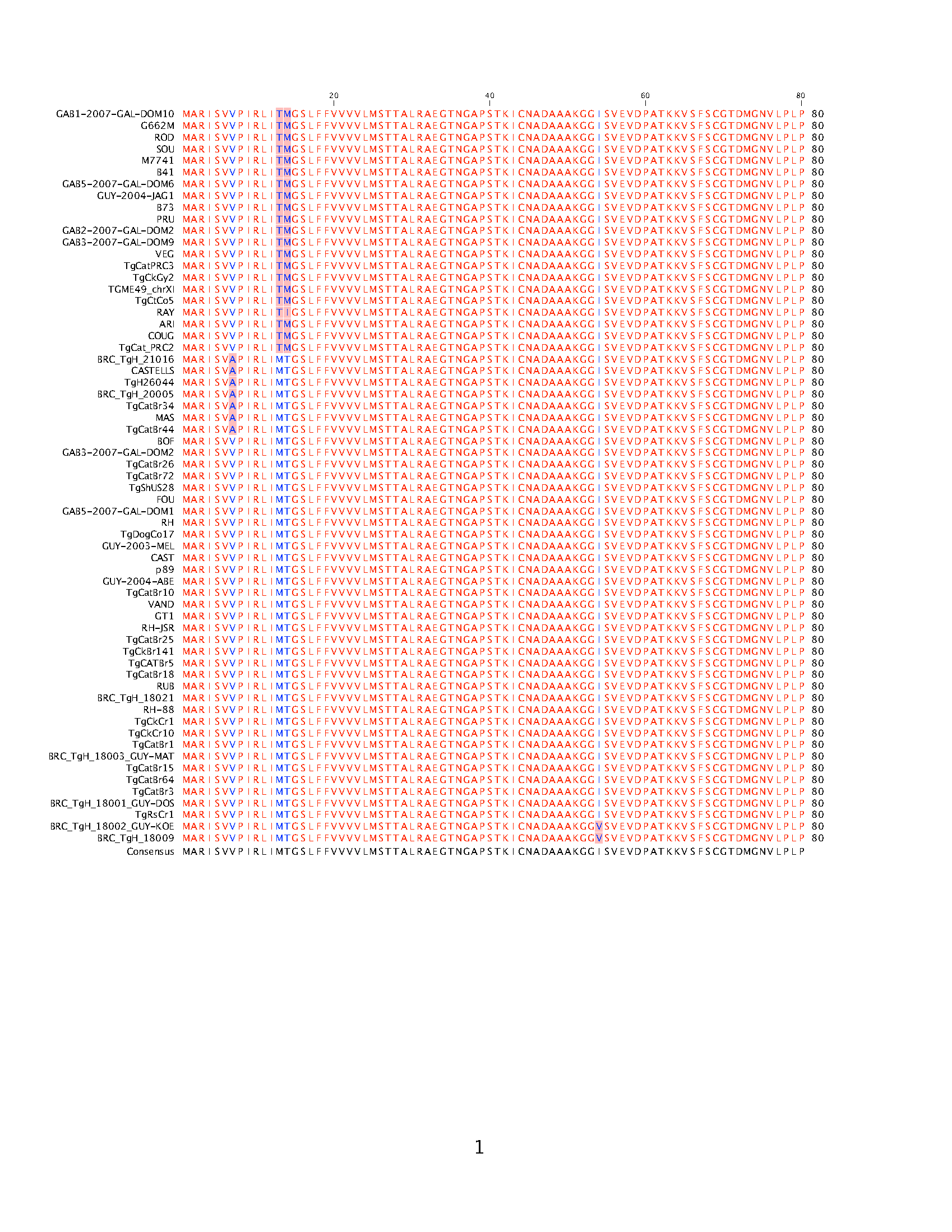


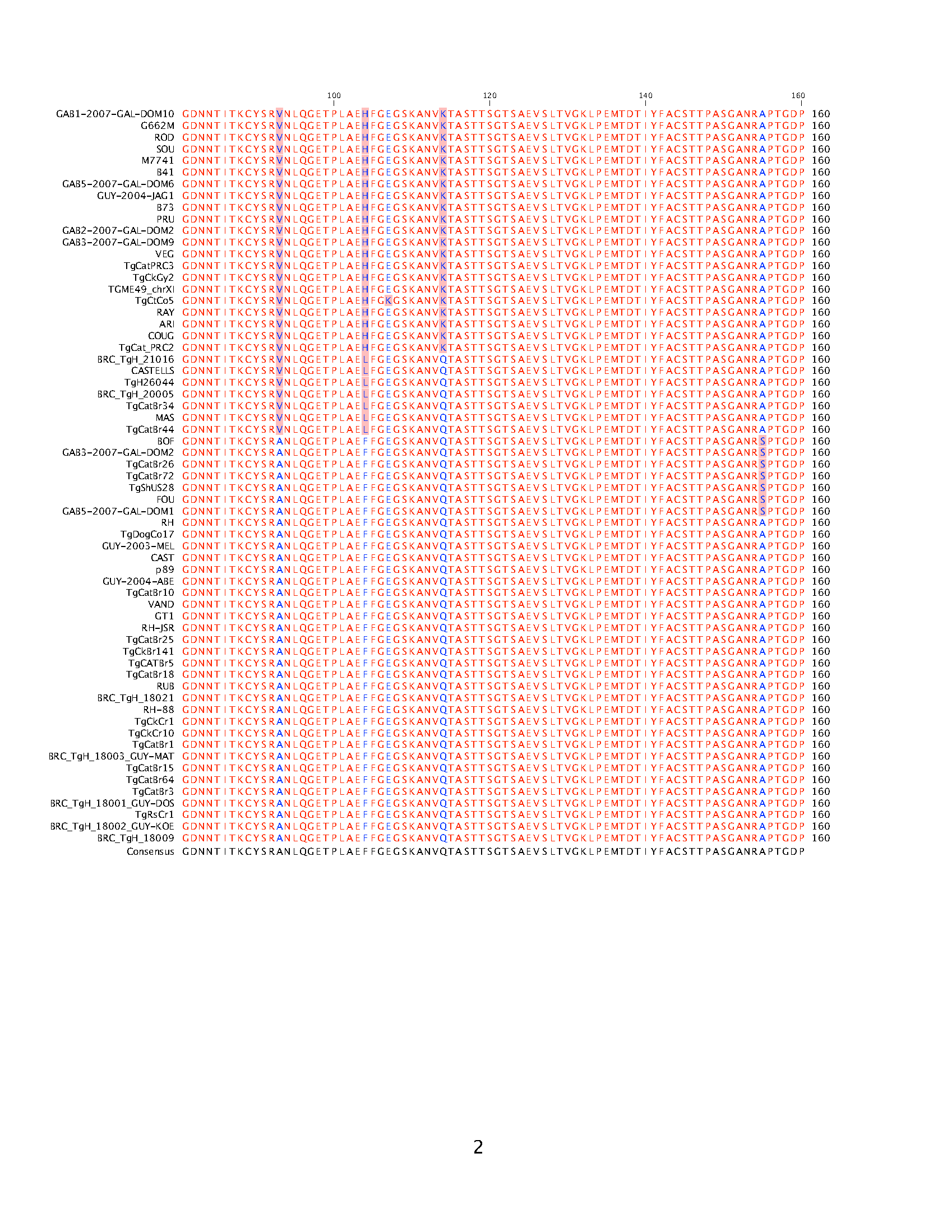


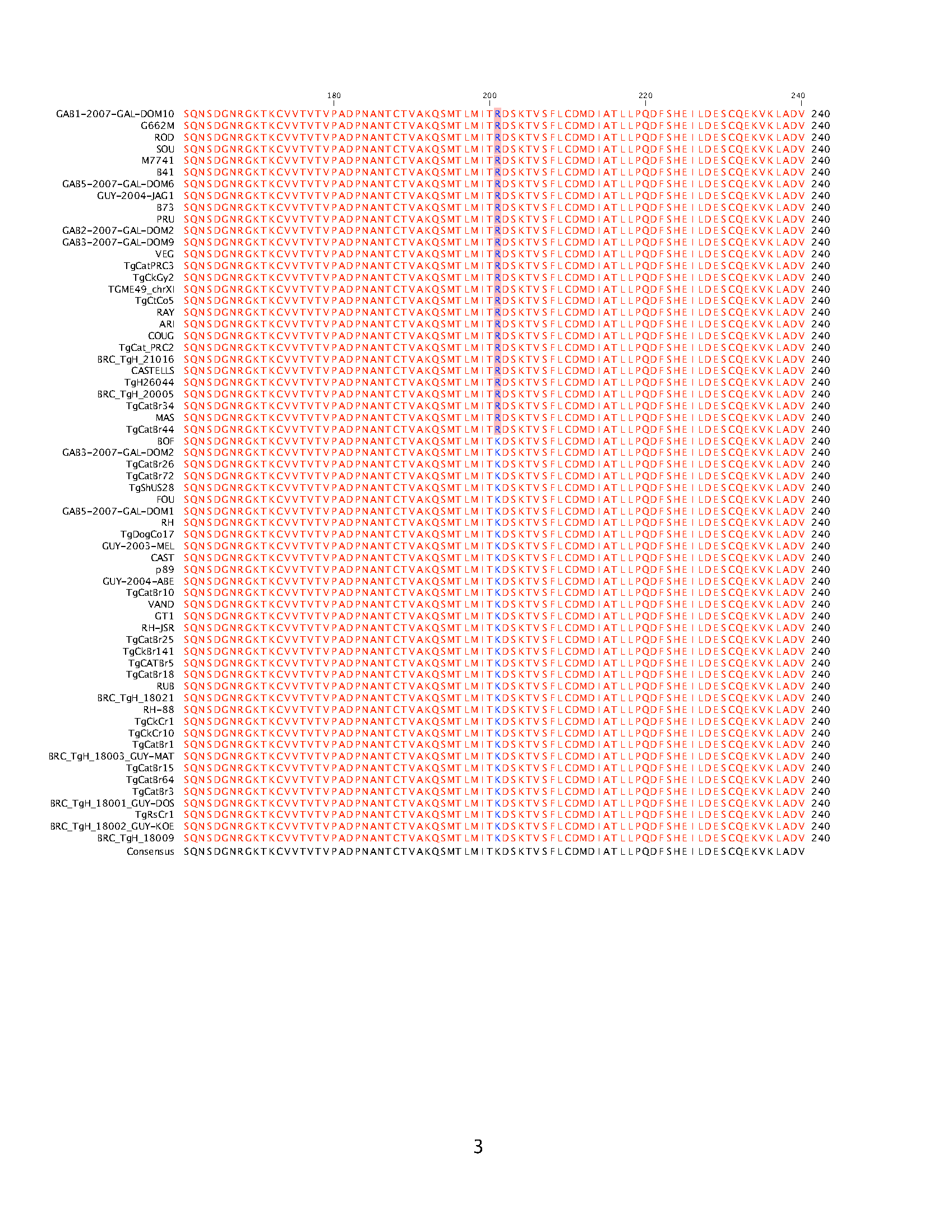


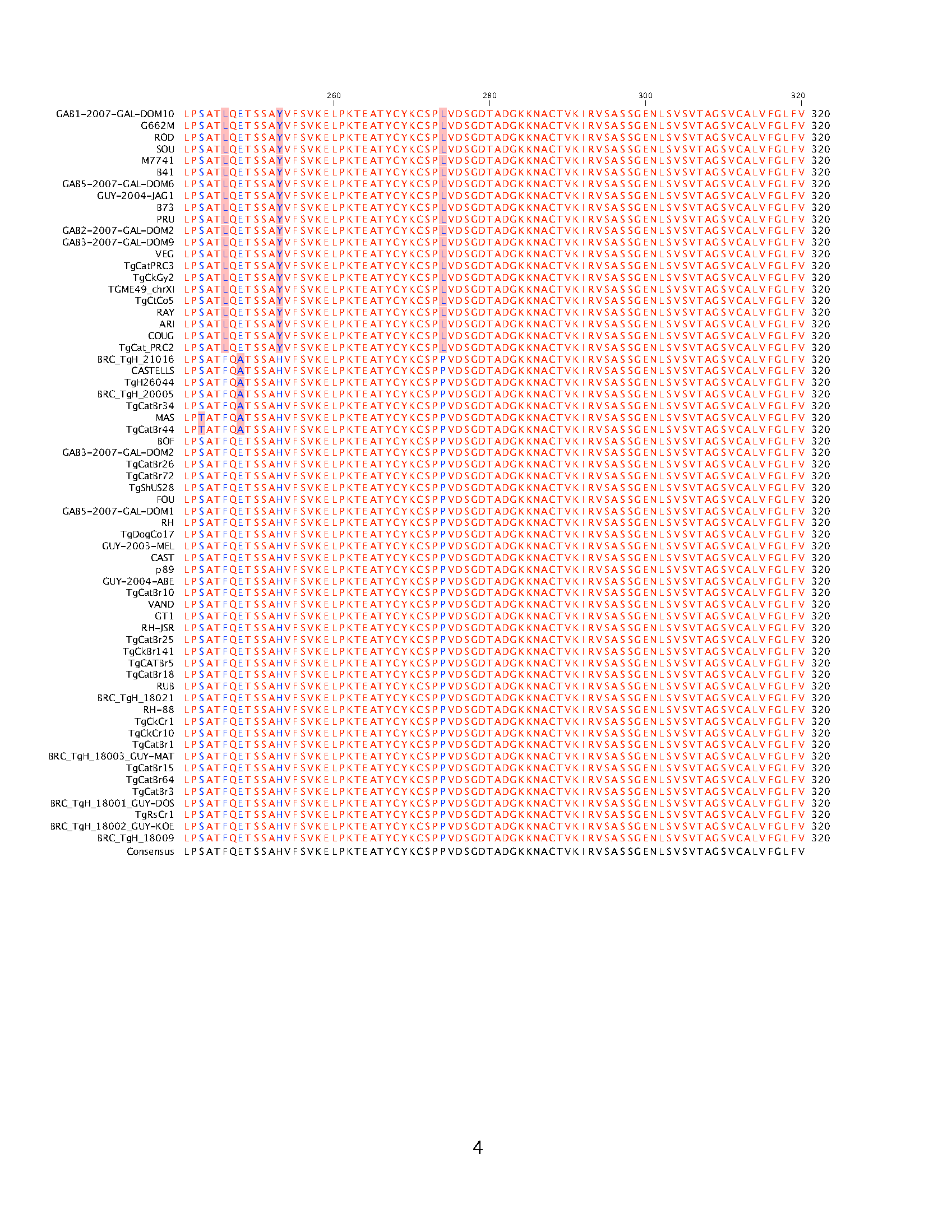


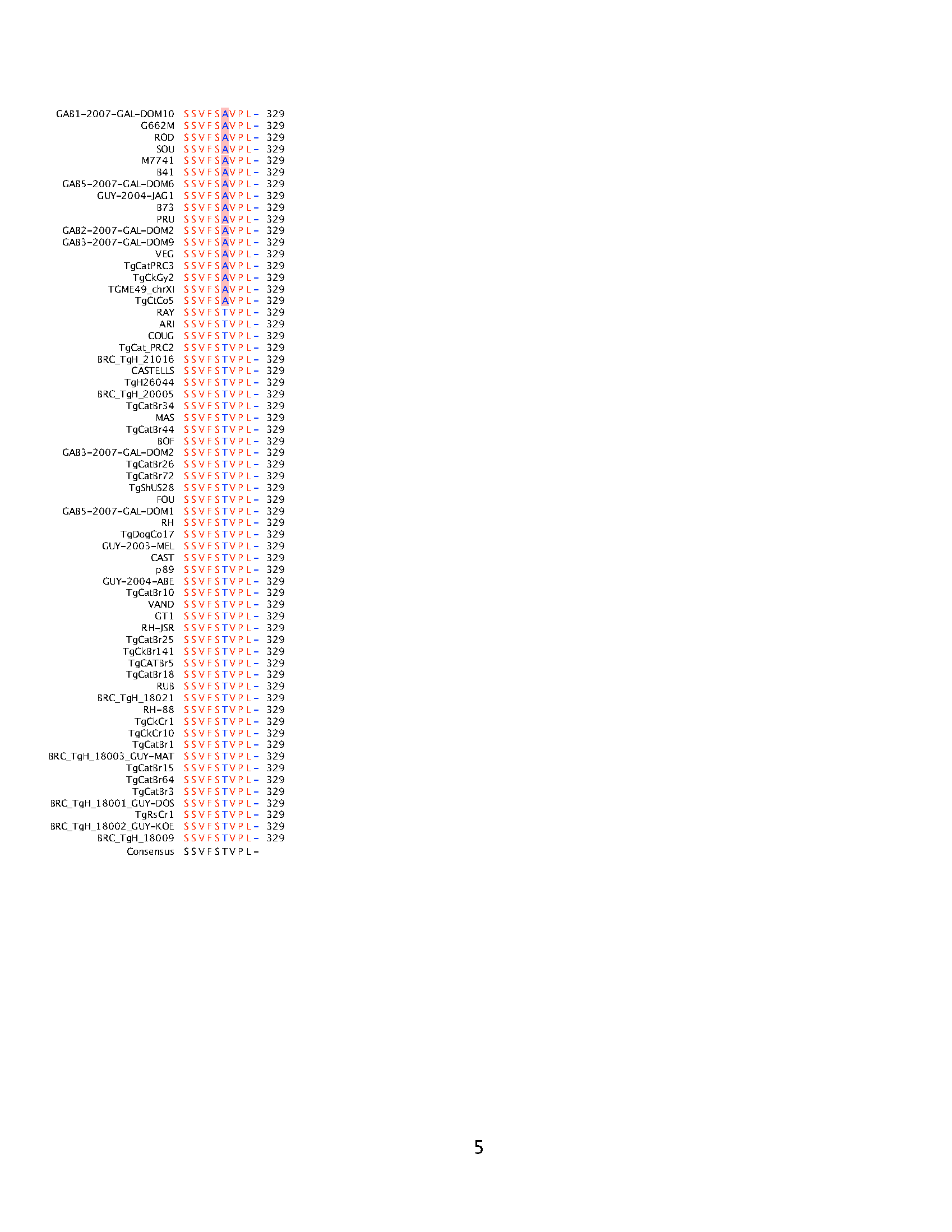

Supplement: Supplementary file 1 — Supplement [file 41541_2017_24_MOESM1_ESM.docx]
